# Proposed dense variational autoencoder model integrated with contrastive learning for foot ulcer classification

**DOI:** 10.1038/s41598-025-34225-7

**Published:** 2026-01-02

**Authors:** Gunjan Shandilya, Sheifali Gupta, Deepali Gupta, Sapna Juneja, Krishnaraj Chadaga, Ali Nauman, Abeer A. Al-Masri

**Affiliations:** 1 Chitkara University Institute of Engineering and Technology, Chitkara University, Rajpura, Punjab India; 2 Krishna Institute of Engineering & Technology (KIET), Ghaziabad, Delhi NCR, Uttar Pradesh, India; 3 Manipal Institute of Technology, Manipal Academy of Higher Education, Manipal, India; 4 School of Computer Science and Engineering, Yeungnam University, Gyeongsan-si, Republic of Korea; 5 Department of Physiology, College of Medicine, King Saud University, Riyadh, 11451 Saudi Arabia

**Keywords:** Diabetic foot ulcer (DFU), Deep learning (DL), Variational autoencoder (VAE), Contrastive learning (CL), DenseNet121, Medical image classification, Automated DFU detection, BYOL (Bootstrap your own latent), Computational biology and bioinformatics, Diseases, Engineering, Health care, Mathematics and computing, Medical research

## Abstract

The most serious complication of diabetes, Diabetic Foot Ulcer (DFU), can result in chronic infection, damage to tissues, and even amputation if not identified in a timely way. It is even more dangerous for disabled people. Accurate and timely diagnosis is therefore essential for improved patient outcomes. However, it is difficult to perform a manual assessment of DFU images due to variations in the textures of ulcers, light effects, and severities. Herein is presented an enhanced deep learning framework DenseNet121 with Variational Autoencoder and Contrastive Learning (DenseVAE-CL) which incorporates DenseNet121 to extract robust features and integrates a Variational Autoencoder together with Contrastive Learning to enhance representation discrimination. The model was trained and tested on 2,673 publicly available images from the Kaggle DFU dataset, which was divided into training, validation, and testing subsets in a ratio of 80:10:10, respectively. DenseVAE-CL emerged as the best performer with an accuracy of 99.6%, precision of 99.5%, recall of 99.4%, specificity of 100%, sensitivity of 98.9%, an F1-score of 99.5%, and 0.99 Area Under the Receiver Operating Characteristic Curve (AUC-ROC). Five-fold cross-validation further demonstrated stable generalization with a mean accuracy of 99.4 ± 0.2%. Moreover, calibration analysis confirmed the model’s reliability since a mean confidence of 0.9031 for abnormal cases and 0.9692 for normal cases results in an expected calibration error of 0.0457. Visualization through Grad-CAM and VAE residual heatmaps revealed sharp localization of ulcer regions, hence improving interpretability.

## Introduction

DFU is a serious complication of diabetes mellitus that affects millions of patients worldwide. The effect of prolonged hyperglycemia is neuropathy and peripheral artery diseases that worsen wound healing and risks of infection, which eventually leads to the formation of foot ulcers^1^. DFUs are a significant health issue they pose to the world at large as it is estimated that over 34% of diabetic patients experience it in their lifetime. Advanced ulcers can develop osteomyelitis and can even lead to amputation of lower limbs in case of not timely treatment^2^. Clinical reports indicate that almost 50% of the patients who are amputated on the basis of DFUs succumb in five years. DFU management takes a major toll of healthcare, where the patients take longer periods of hospitalization, repeated visits in the outpatient unit and consume a lot of finances. In addition, DFU complications increase the susceptibility of cardiovascular and kidney diseases, which make patient management more difficult^3^. Early and correct identification of DFUs has been a significant problem in clinical practice. Clinician assessment is subjective and time consuming in achieving manual inspection and is more likely to experience inter-observer variability particularly in large patient groups^4^. Therefore, there is a strong need for automated, reliable, and objective DFU detection systems to support early diagnosis and improve patient outcomes.

The last breakthrough in the field of deep learning (DL) resulted in the creation of the automated diagnostic tools that show a high level of performance in a range of medical imaging assignments. These models are based on big data to assist clinicians in identifying DFUs earlier to help them treat them in time. Older approaches to machine learning (ML) that use handcrafted features extraction are incapable of generalizing and are prone to failure in various imaging conditions^5^. Moreover, lack of adequate and biased training data may lead to ineffective model performance and unreliable prediction. Conversely, DL architectures have the capability to extract hierarchical representations with large-scale data automatically, which allows them to provide more accurate diagnostics and earlier identify DFUs^6^.

Regardless of this development, a majority of current DFU classification works rely on traditional convolutional neural networks (CNNs) or transfer learning models that suffer limitations in their ability to generalize among the different textures of ulcers, lightings, and levels of severity. The challenges of this model are that they overfit because of limited diversity in data, they are not interpretable and using handcrafted augmentations. To overcome these difficulties, this research paper presents a hybrid generative-contrastive DL model, including DenseVAE-CL, that combines the feature regularization capability of Variational Autoencoders (VAE) and the discriminability of representations of Bootstrap Your Own Latent (BYOL)-based Contrastive Learning (CL). This framework aims at enhancing generalization, interpretability, and calibration reliability using DFU classification with an end-to-end framework that allows one to fill the gap between purely discriminative CNN models and generative-contrastive frameworks.

This study aims to use DL methods to detect and classify DFUs one of the most severe complications of diabetes mellitus automatically. The suggested DenseVAE-CL model is a refined version of DenseNet121, which involves VAE and CL to obtain a better feature extraction, representation learning, and classification accuracy. VAE is a dimensionality reduction and latent feature enrichment module, and it provides important ulcer-related structures and enhances generalization between patient datasets. CL component strengthens the concept of feature discrimination and makes inter-class separation and intra-class variation as large as possible, which enhances robustness and reliability. DenseNet121 with the additional Squeeze-and-Excitation (SE) blocks further optimize the processes of recalibration of features, identify the most informative channels as the classification process takes place. In combination with each other, these elements help the model to identify healthy and ulcerated areas in a highly accurate fashion, contributing to the early and consistent detection of DFU.

Unlike the biomedical hybrids that existed before, using either VAE or contrastive learning in a separate manner, the proposed DenseVAE-CL optimizes jointly for reconstruction and discriminative embedding consistency. This synergy enables the network to capture subtle structural differences between healthy and ulcerated skin, addressing intra-class variability often disregarded in prior works. The model illustrates high accuracy in classification and robustness, hence making it suitable for future integration into decision-support and telemedicine-oriented screening workflows, subject to further validation.

The main contributions of this study are:

1. A novel deep learning framework, DenseVAE-CL, is proposed by integrating a fine-tuned DenseNet121 enhanced by Squeeze-and-Excitation (SE) blocks with Variational Autoencoder (VAE) and BYOL-based Contrastive Learning. This hybrid design enhances both feature extraction and class separation, leading to superior diagnostic accuracy in binary foot ulcer classification tasks.
2. Advanced multi-step image preprocessing strategy is employed, including resizing, normalization, noise reduction, gaussian smoothing and affine transformations, to enhance model robustness and improve generalization on diverse foot image data.
3. The contrastive learning strategy, implemented via BYOL, enhances the model’s discriminative ability by learning invariant features that differentiate healthy and ulcerated foot conditions, thereby reducing misclassification and boosting detection reliability.
4. This study enhances the standard DenseNet121 architecture by incorporating SE blocks, which perform channel-wise feature recalibration. This integration allows the model to focus on the most informative and class-relevant features while suppressing irrelevant or noisy activations.
5. The VAE component learns compact and meaningful latent representations, enabling the generation of synthetic foot images that improve model generalization and robustness. This supports training with more diverse data and addresses overfitting.

This article is divided into six sections that cover the whole research study. The introductory part, Sect. 1, presents background, research motivation of the study. Section 2 contains the Literature Review that analyzes existing DFU detection research. Section 3 covers the methodology part that will introduce the proposed framework. Section 4 will cover results about the implementation of the proposed approach, experimental outcomes, and performance metrics. Section 5 includes the State of Art comparison and Sect. 6 summarizes the essential findings while assessing their importance and proposing future research strategies with regard to the study objectives and the gaps found in previous works.

## Literature review

DFU classification has emerged as one of the most important research areas in the field of medical AI, and several ML and DL methods have been proposed with the scope of enhancing diagnostic accuracy and clinical decision support. Several of the earlier models illustrated the potentiality of the deep networks in the identification of DFUs. Authors of^7^ proposed a hybrid framework incorporating CNN, ViT, and SNN on the DFU2021 dataset, comprising 5955 images divided into four classes, where the model attained an accuracy of 93.1% and F1-score of 95.2%, hence demonstrating the capability of the ViT in detecting spatial patterns in DFU imagery. Similarly, authors of^8^ employed the Sparrow Search Optimization algorithm to optimize Inception-ResNet-v2 on the Kaggle DFU dataset comprising 844 images and realized an improved accuracy of 99.29%, whereas^9^ got an accuracy of 98.97% through EfficientNet on the same dataset, hence demonstrating the efficiency of compound scaling for medical image classification.

The researchers in^10^ expanded EfficientNet’s compound scaling on the DFU2021 dataset, achieving 98% accuracy for infection and 99% for detecting ischemia. Methods based on ensembles have been investigated as well:^11^ employed Ensemble CNNs on DFU2021 (1459 images), attaining 73% accuracy for infection classification and 90% for ischemia classification, even with the variability in the dataset. Conventional ML-based approaches have also been explored. For instance^12^, utilized the Light Gradient Boosting Machine (LightGBM) integrated with SHAP explainability on a hospital dataset comprising 618 images from four categories, achieving a recall of 87.1%, precision of 86.3%, and ROC-AUC of 0.90. These studies highlight the transition from manual ML techniques to more advanced DL frameworks for DFU detection.

Thermographic and hybrid models based on CNN have further assisted in the analysis of DFU. Study in^13^ used VGG19 on patient thermograms grouped into four categories, achieving 95.08% accuracy. Authors in^14^ proposed ScoreDFUNet, a deep CNN that was trained on combined DFUC2020, hospital, and Kaggle data (1944 images, four categories), achieving 95.34% accuracy and proving that CNN-based models are competent for diagnostics of DFU. In the research presented in^15^, the authors propose a hybrid Swin Transformer Efficient Multi-Scale Attention network with feature fusion for DFUC2021 images (5955, two classes) and achieve 78.79% accuracy, 81% precision, 79% recall, and 80% F1-score, stating the growing role of attention mechanisms in DFU analysis. In addition^16^, introduced a CNN model E-DFU-Net, trained on DFU2021 (1459 images, two categories), reaching 94.7% accuracy and AUC of 0.947, while^17^ introduced Swin DFU-Net, a Transformer–CNN hybrid, trained on 5890 images, reaching 96.52% accuracy and 96.55% F1-score.

Recent CNN-based models have explored better architectures and more diverse data. The DFU_SPNet model proposed in^18^ used three parallel convolutional blocks with varying kernel sizes to distinguish between DFU and normal skin. Using the DFUNet dataset of 1679 hospital images, DFU_SPNet achieved 96.4% of accuracy, 92.6% of precision, 98.4% of recall, and an AUC of 0.974. Research in^19^ combined texture and RGB features for DFU classification, achieving AUCs of 0.981 and 0.995 for ischemia classification and 0.820 for infection detection-demonstrating advantages of multiple feature fusion. Research^20^ presented a four-branch CNN to alleviate the problem of vanishing gradients, achieving a 95.8% F1-score on the DFU dataset. Similarly^21^, explored traditional ML for thermographic analysis of DFU without using SIFT, SURF, or BOF and managed to acquire accurate temperature mapping straight from the foot thermograms.

Recent works have focused on improving generalization with model ensembles and attention-driven learning. The work in^22^ presented five modified CNNs for DFU classification on 8250 augmented images with different contrast and resolutions, which resulted in a mean accuracy of 95.04% and a Kappa score greater than 91.85%. DFU-VGG^23^ was an augmented version of VGG19 with dense layers and batch normalization, analyzing 8250 DFU images to attain an accuracy of 93.45% and a Kappa index of 0.892. ACTNet^24^ proposed an asymmetric convolutional transformer for classifying four classes of DFU diagnosis, achieving an F1-score of 0.593 and an AUC of 0.824 on the DFUC2021 test dataset. Furthermore, the concept of federated learning in DFU detection was explored in^25^, presenting the Federated Averaging P2P and Federated SGDP2P algorithms, both attaining a similar convergence as that of centralized methods but maintaining data privacy.

In summary, recent trends highlight a very clear shift in the use of conventional ML systems for DFU classification towards more Transformer-based architectures and federated systems. While CNN-based EfficientNet and VGG19 provided excellent baselines, hybrid architectures like Swin DFU-Net and combinations of CNN-ViT-SNN have achieved better generalization and discriminative performance. Future trends point to the use of federated learning with multimodal imaging and self-supervised contrastive methods, including loss and regularization terms, as promising future avenues towards more robust clinical interpretability in DFU diagnosis.

## Proposed methodology

**Fig. 1:**
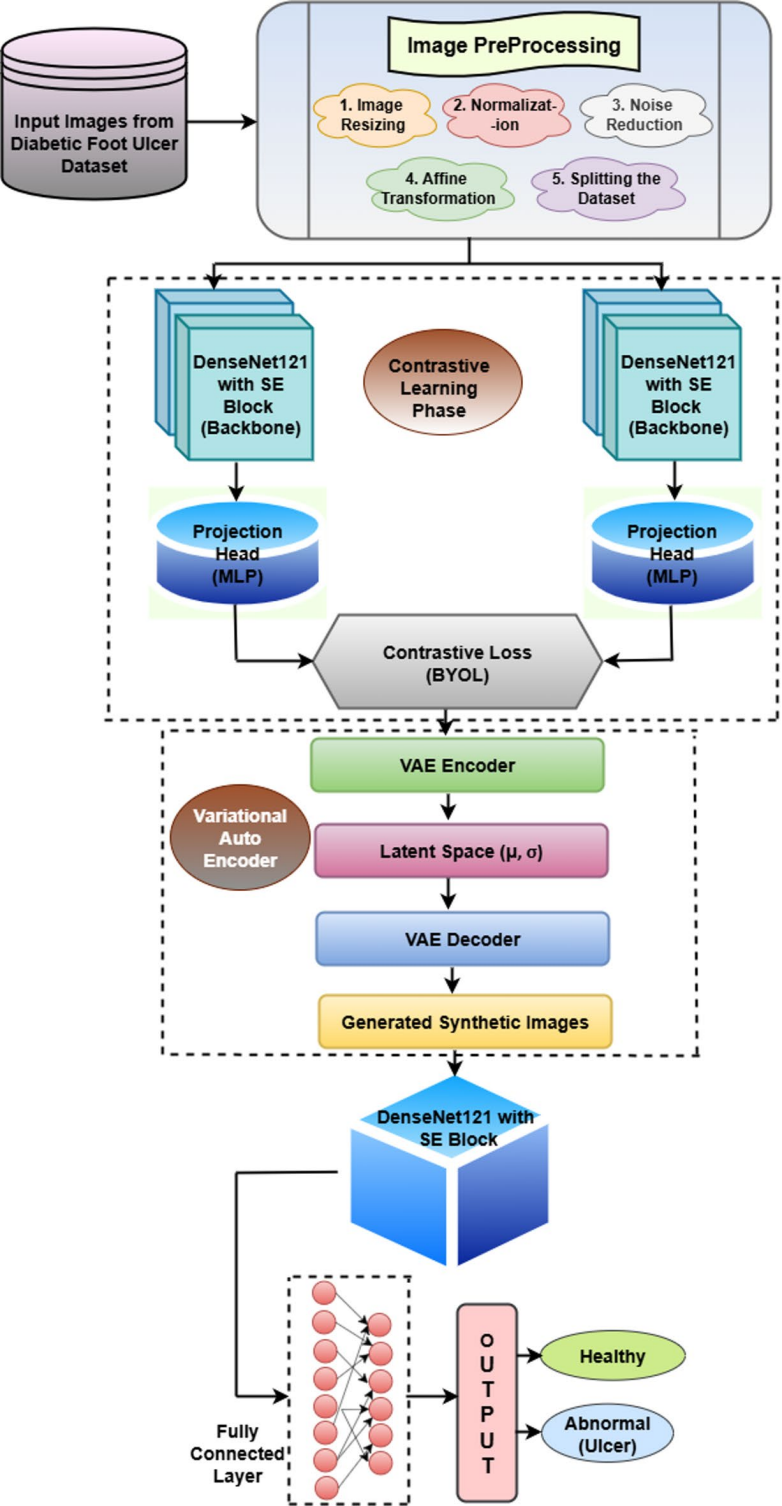
Proposed methodology.

An all-encompassing description of a DL detection model that uses foot image data to identify DFU is shown in Fig. 1. It can be seen that the beginning phase of image preprocessing applies three changes to the images: image size and normalizes them for performance enhancement while removing unneeded noise. The dataset receives additional affine changes which improve both the robustness and generalization capability of the model. The dataset undergoes a subsequent procedure that divides it into areas for training purposes, validation and testing purposes. The CL phase implements a DenseNet121 architecture with SE block as its central model component. The architectural design enables the model to discover vital image features. The DenseNet121 output passes through a projection head (MLP) to get converted into a lower-dimensional space before comparison takes place. The BYOL self-supervised learning method helps the model gain valuable representation comprehension by comparing augmented images, facilitating feature extraction without the need for labeled data. VAE features an encoder component that converts images into latent space representations characterized by mean (µ) and standard deviation (σ).

The VAE includes a decoder that utilizes latent variables to regenerate images. The method produces new synthetic pictures to increase the dataset size. Secondly, the VAE produces new synthetic images but the DenseNet121 model with SE block processes them to enhance the learned features. The model concludes its process by performing binary classification which separates images between healthy and abnormal (ulcer) categories. Finally, the model performs binary classification, distinguishing between two categories: healthy and abnormal (ulcer). Through the combined method of CL and synthetic image generation the model becomes better able to grasp important features while achieving enhanced detection performance. Using real images together with synthesized synthetic images in training data enables better generalization of the model while producing precise predictions which aids in early diagnosis of DFU in medical facilities.

### Dataset description

The DFU Dataset^26^ is a publicly available dataset on Kaggle that gathers foot imaging data for the recognition of DFUs among various categories. The dataset comprises patient-derived images collected in Iraq. Ethical approval and written informed consent were obtained by the Al-Nasiriyah Diabetic and Endocrinology Center at the time of data collection. The dataset does not contain any personally identifiable patient information. All methods in this study were carried out in accordance with the relevant guidelines and regulations governing the use of publicly available medical image data. There are 2673-foot images in this dataset, which is divided into normal and abnormal (ulcer) varieties. The DFU dataset^26^ does not include unique patient identifiers, therefore, all splits were performed at the image level using an 80:10:10 ratio for training, validation, and testing, respectively. The DFU data provides essential value for researchers working on automatic DFU detection systems since it supports building predictive algorithms for early diagnosis and treatment. Because the dataset contains clear hierarchical organization and its labeled classification structure, it can be used within supervised learning frameworks for detecting healthy foot regions from ulcerated areas.

**Fig. 2:**
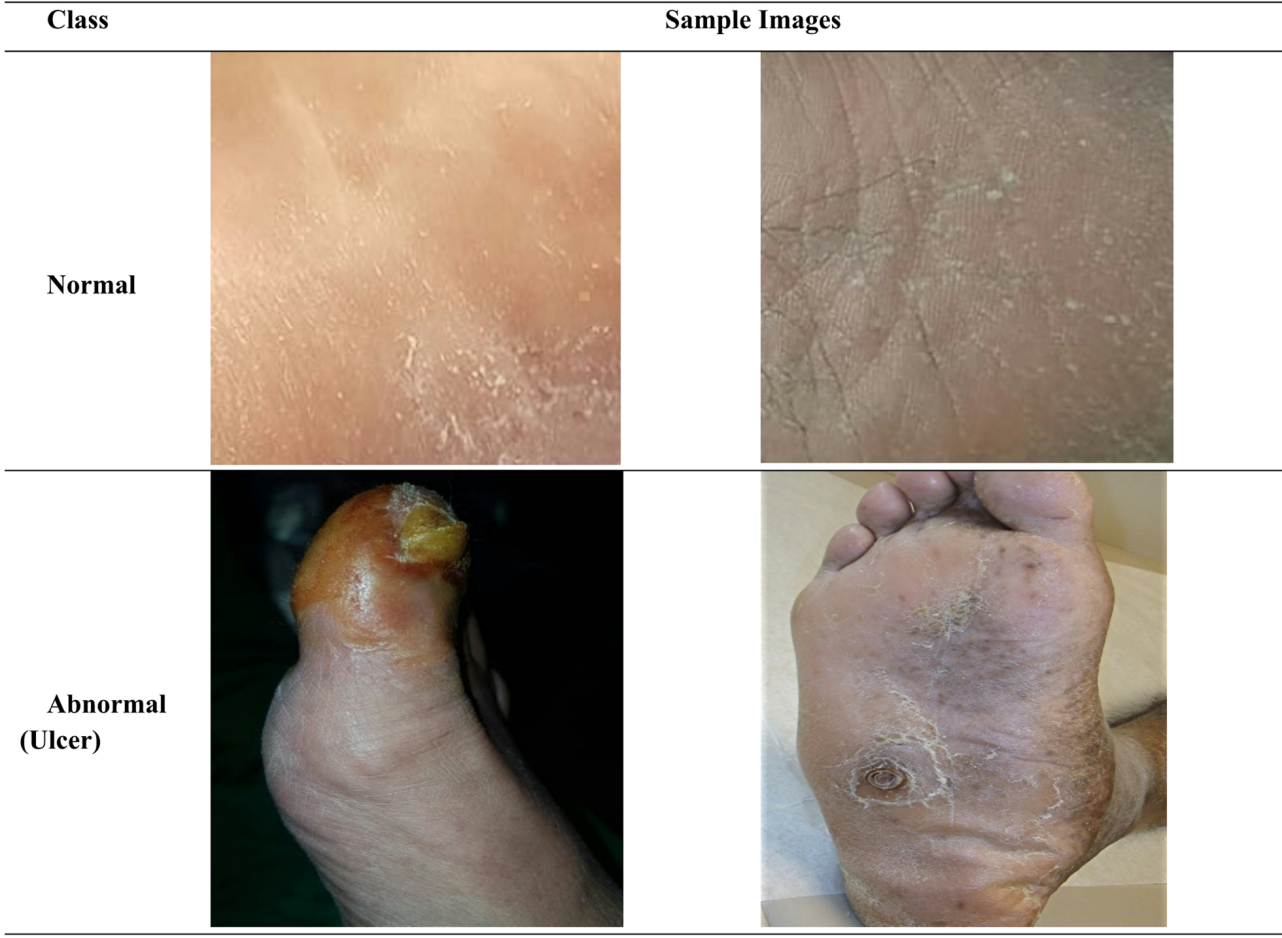
Sample images from Dataset^26^.

The DFU Dataset hosts essential information on Kaggle as a valuable research tool for those working in medical imaging fields along with disease classification. The users need to observe ethical rules and cite the original origin of the dataset during research activities or development. Figure 2 showcases the sample images from DFU dataset.

### Preprocessing

**Fig. 3:**
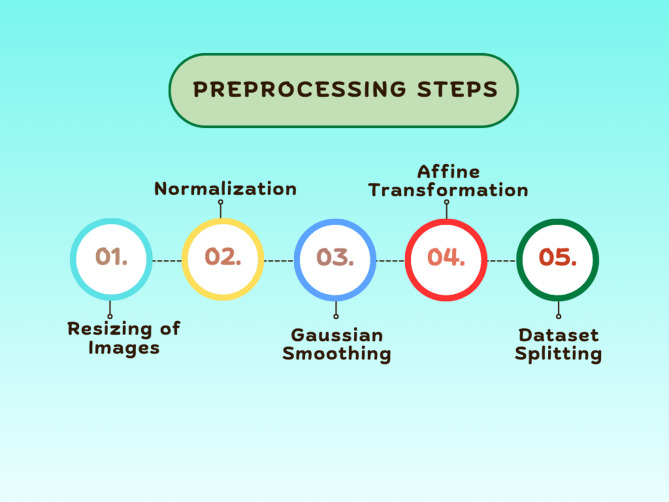
Overview of image preprocessing pipeline for DFU detection.

This section discusses various preprocessing techniques employed in this study. Figure 3 illustrates the major steps of the image preprocessing task, an important phase in computer vision for improving quality and maintaining consistency before inputting data into a DL model. It emphasizes five most important techniques: resizing the image, normalizing the image, reducing noise, performing affine transformation, and splitting the dataset. Image resizing ensures that all images have the same size for simplification of processing by CNNs. Mathematically, a size change of an image from (H, W) to (H′, W′) is performed by interpolation, in which the value of a pixel at a new location is calculated from the original coordinates.

The images are adjusted to a consistent dimension of 224 × 224. Normalization adjusts pixel values to a defined range, usually [0,1] or [-1,1], by applying the formula below to enhance model convergence:

1 $$\:{I}_{normalized}=\:\frac{I-\mathrm{m}\mathrm{i}\mathrm{n}\left(I\right)}{ma\left(I\right)-\mathrm{min}\left(I\right)}$$

Reducing noise is a crucial step that removes unwanted variations and artifacts in images, as these can otherwise impair model performance. Gaussian Smoothing is a common technique that employs a Gaussian filter to soften the image, defined by:

2 $$\:G\left(x,y\right)=\:\frac{1}{2\pi\:{\sigma\:}^{2}}{e}^{-\frac{{x}^{2}+{y}^{2}}{2{\sigma\:}^{2}}}$$

The filtered image is obtained by convolution:

3 $$\:({I}^{{\prime\:}}\left(x,y\right)=\:\sum\:_{i=-k}^{k}\sum\:_{j=-k}^{k}I\left(x-i,\:y-j\right)G(i,j)$$

Here G(i, j) is the Gaussian kernel.

**Fig. 4:**
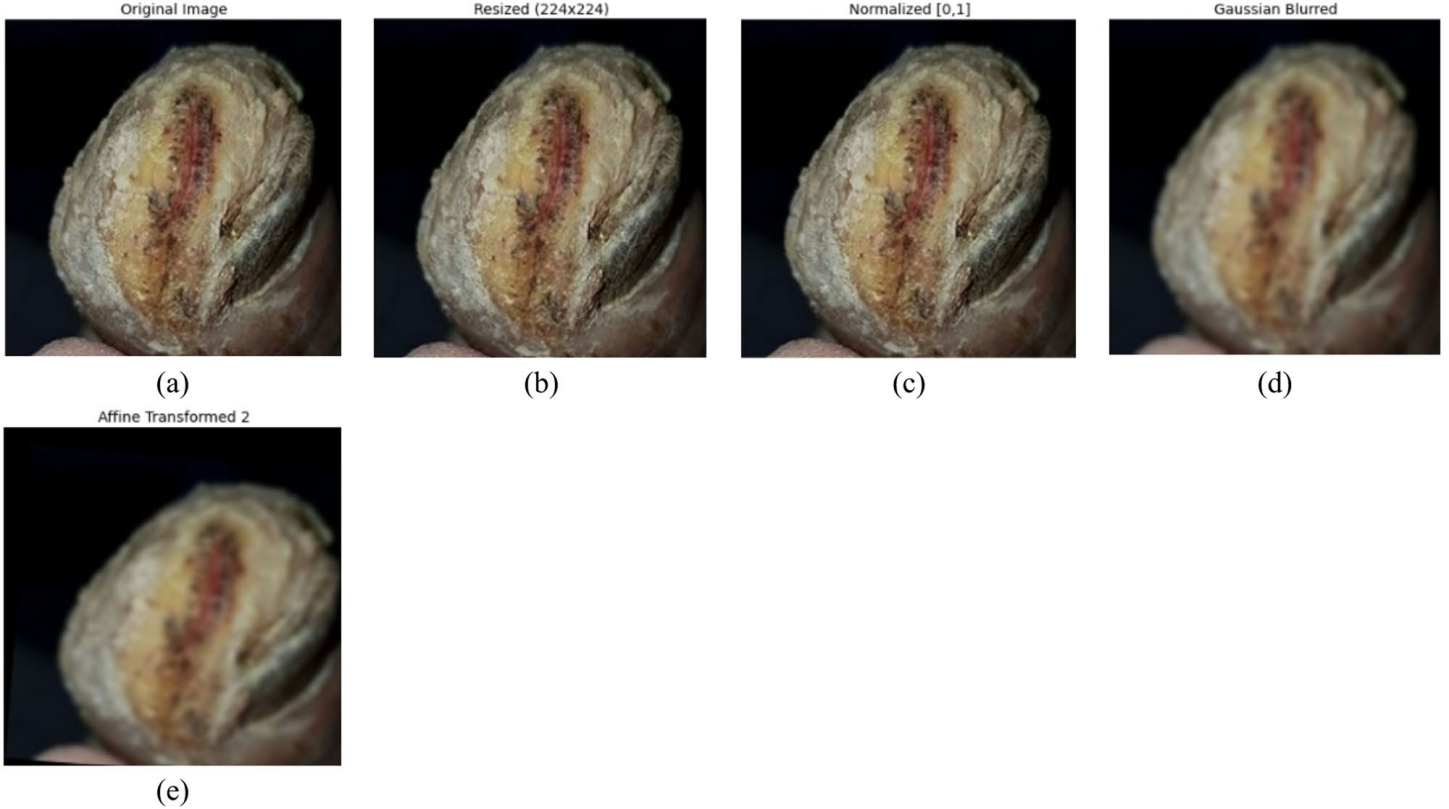
Visualization of preprocessing (**a**) original image (**b**) resized (**c**) normalized (**d**) Gaussian Blur (**e**) affine transform.

Affine Transformation is a particular form of data augmentation method that enables geometric modifications like translation, rotation, and scaling while maintaining parallelism in images. The conversion is expressed mathematically as:

4 $$\:\left[\begin{array}{c}{x}^{{\prime\:}}\\\:{y}^{{\prime\:}}\end{array}\right]=\:\left[\begin{array}{ccc}a&\:b&\:tx\\\:c&\:d&\:ty\end{array}\right]\left[\begin{array}{c}x\\\:y\\\:1\end{array}\right]$$

where (x′, y′) are the transformed coordinates, (x, y) are the original coordinates, and tx, ty are the translations. The transformation matrix allows for rotation, scaling, and translation. Each of the 2,673 images underwent two randomly chosen transformations. Due to these augmentations, the image number increased to 5346. Finally, splitting of the dataset is carried out. This step ensures that the model will be trained, validated, and tested on different sets of images to avoid overfitting and to estimate the efficiency of generalization. Usually, the dataset is split into training, validation, and test sets in a ratio such as 80%-10%-10%, which in a mathematical expression would look like:

5 $$\:{D}_{train}=p\:X\:d$$

6 $$\:{D}_{validation}=q\:X\:d$$

7 $$\:{D}_{test}=r\:X\:d$$

where p + q + *r* = 1. The training set then contains 4277 images, the validation set contains 535 images, and the test set contains 534 images after the split. These steps in preprocessing significantly enhance the accuracy and resilience of models by normalizing the input images and reducing noise while increasing available data for better generalization of the model. Figure 4 shows the effect of each preprocessing method on the original image.

**Fig. 5:**
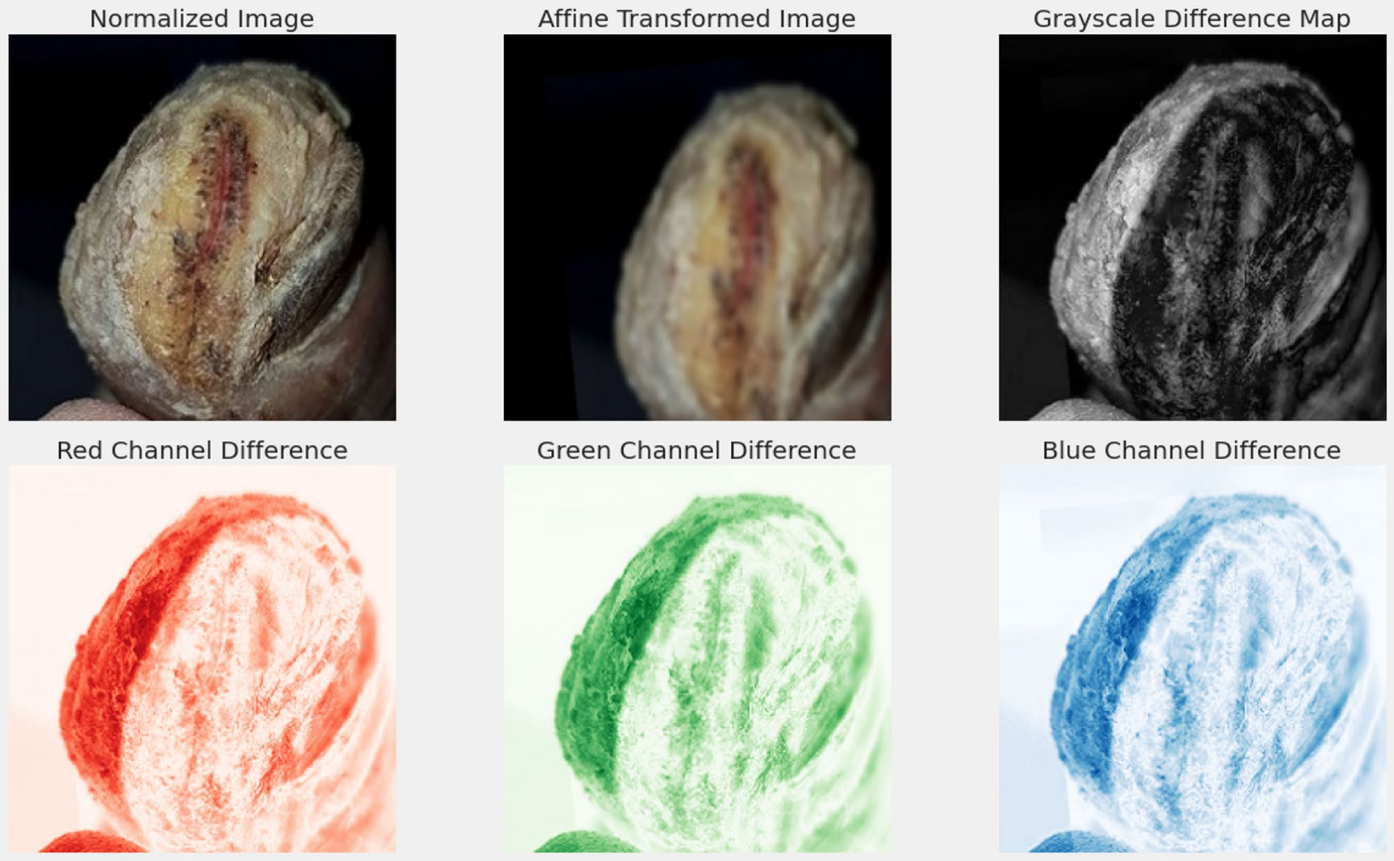
Preprocessing and spectral difference maps of DFU images.

The first image in Fig. 5 represents the normalized input, where the pixel values are scaled within the range of [0,1] to make all the samples consistent for better model convergence. The second image represents the result of an affine transformation; such a geometric transformation involves rotation, translation, and scaling of the input image, introducing more spatial variability that helps the model generalize better.

The third image is a grayscale difference map highlighting the structural changes between the original and transformed images, providing information on the regions most affected by augmentation. The bottom row further explores these differences by decomposing them into individual color channels. Difference maps of red, green, and blue channels outline the spectral variations resulting from the transformation, thus enabling a more accurate localization of anomalies. This channel-by-channel analysis will enhance color-based feature extraction for more robust and accurate classification of ulcerous and non-ulcerous regions of the foot during training.

### DenseNet121 with SE block

**Fig. 6:**
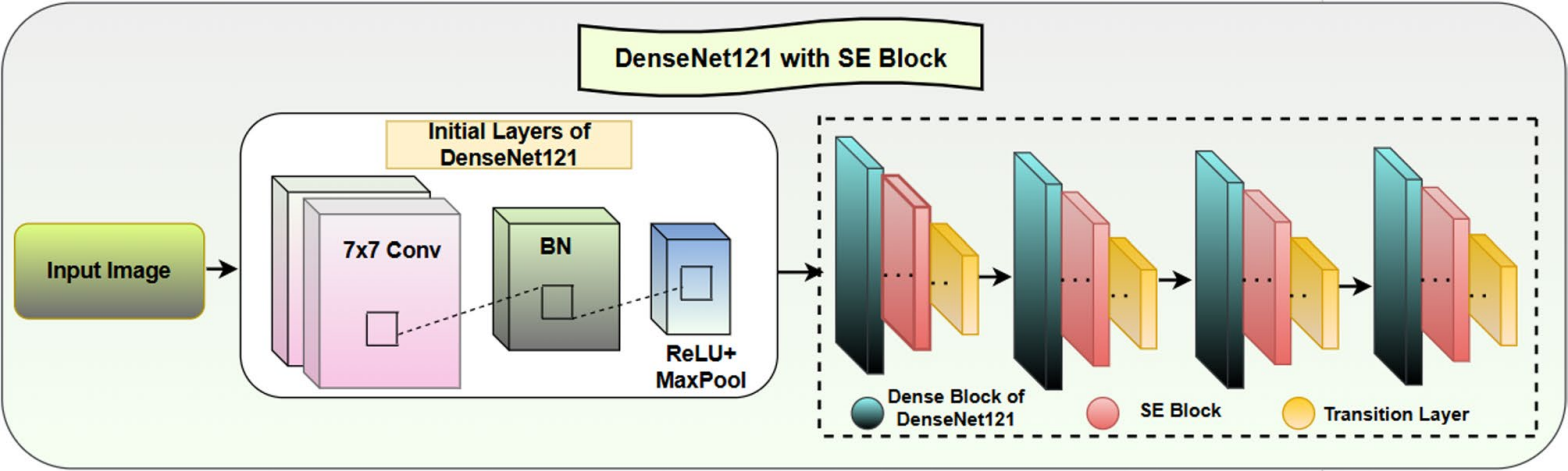
DenseNet121 with SE block architecture.

The architectural design in Fig. 6 combines SE Blocks and DenseNet121 to enhance feature representation and improve model performance. Because of its dense connectivity nature, DenseNet121 is able to propagate information very effectively across features while minimizing parameters to sustain the efficiency of classification. The model obtains superior performance through SE Blocks that allow it to pay attention to crucial features while minimizing irrelevant features. From Fig. 6, it can be seen that in DenseNet121 model, SE blocks are inserted after each Dense Block and before every Transition Layer within the DenseNet121 backbone. This placement enables the network to perform channel-wise recalibration after feature concatenation, allowing the model to emphasize ulcer-related channels before spatial down-sampling. The inclusion of SE blocks enhances representational selectivity and suppresses redundant activations, improving discriminative power with minimal parameter overhead.

DenseNet121 executes its first convolutional layers on an input image through a 7 × 7 convolutional layer that expands its receptive field to recognize basic elements such as edges and patterns and textures. The network applies Batch Normalization (BN) for distribution normalization, after which the training becomes more stable, leading to faster convergence. The combination of a ReLU activation function and MaxPooling brings both non-linear transformation and dominant feature extraction to the system. The convolution operation is calculated as:

8 $$\:Y\left(i,j\right)=\:\sum\:_{m=0}^{M-1}\sum\:_{n=0}^{N-1}X\left(i-m,\:j-n\right).W\left(m,n\right)$$

Here, the input feature map is given by X(i, j), the convolutional kernel is given by W(m, n), whereas M and N represent the kernel dimensions. The BN operation is given as:

9 $$\:{\widehat{x}}_{i}=\:\frac{{x}_{i}-\mu\:}{\sqrt{{\sigma\:}^{2}+\epsilon}}$$

Here, x_i_ is the input, and ε is a small constant for numerical stability. After a few initial layers, the network goes through several Dense Blocks, where each layer receives input from all the previous layers in a feed-forward manner. Unlike the traditional CNNs, which rely on a linear connectivity pattern, DenseNet121 establishes direct connections between all layers, making the reuse of the features learned earlier possible. This promotes the flow of the gradient, reducing the problem of vanishing gradients and also reducing the number of parameters compared with traditional deep networks.

To enhance the feature selection process, SE Blocks are incorporated into the Dense Blocks. The mechanism of these blocks involves three major steps: Squeeze, Excitation, and Recalibration. During the Squeeze phase, a GAP layer reduces each feature map to one value, capturing the spatial information of the entire image. During Excitation, the two fully connected FC layers use ReLU activation in the first layer and Sigmoid activation in the second layer to compute the per-channel attention weights. During Recalibration, the calculated weights are used to multiply the original feature maps to control channel intensities or to attain specific channel suppression results.

The network implementation through this mechanism selects important features while eliminating additional unnecessary information. The SE block carries out a two-step process:

- Squeeze Operation is given as:

10 $$\:{s}_{c}=\:\frac{1}{H\:\mathrm{x}\:W}\sum\:_{i=1}^{H}\sum\:_{j=1}^{W}{X}_{c}\left(i,j\right)\:\:\:\:$$

- Excitation Operation is given by:

11 $$\:z=\:\sigma\:({W}_{2}\delta\:\left({W}_{1}s\right)$$

In this context, X_c_ (i, j) represents the feature map for channel c, while W1 and W2 are the fully connected layers, and σ signifies the Sigmoid activation function.

Transition Layers serve as bottlenecks between Dense Blocks and reduce the dimensionality and computational load. These layers consist of 1 × 1 convolutions that compress feature maps, followed by Average Pooling to further reduce spatial dimensions. These transition layers are needed to prevent excessive redundancy in features and overfitting and to maintain detailed feature representations. The Transition operation is defined by:

12 $$\:Y=\:\frac{1}{k}\sum\:X$$

k denotes the size of the pooling window. SE Blocks added to DenseNet121 resolve a crucial deficiency that traditional CNNs have due to their inability to identify more and less important features. The ability of the SE Block for channel-wise recalibration provides the network with dynamic adjustment of feature importance, which positively affects generalization and accuracy.

### MLP: multi-layer perceptron

**Fig. 7:**
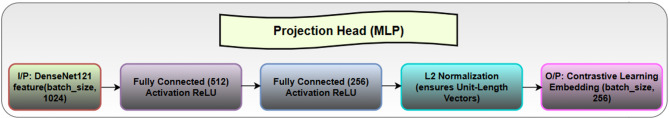
MLP architecture.

The illustration in Fig. 7 displays a Projection Head functioning as a CL^27^ tool for feature embeddings derived from DenseNet121. The projection head handles input feature vectors with dimensions starting from batch_size and finally having 1024 elements each. A ReLU-activated projection head transforms the 1024-dimensional vectors into 512-dimensional vectors which then activates these values using a ReLU non-linear function. Feature dimensions pass through the second FC layer to reach 256 dimensions then activate through ReLU. L2 normalization of feature vectors takes place before output to establish unit lengths which both enhances feature collapse prevention and maximizes efficiency in the similarity evaluations of CL. The output vector of size (batch_size, 256) provides the inputs for contrastive learning embedding operations. A linear transformation and ReLU activation and L2 normalization procedures generate the CL embedding using the network. The process of L2 normalization creates features vectors with unit length:

13 $$\hat{z} = \frac{z}{{\left\| z \right\|_{2} }} = \frac{z}{{\sum\nolimits_{{i = 1}}^{d} {z_{i}^{2} } }}$$

Here $\:\widehat{z}$ is the normalized output vector, z is the original feature vector and d is the feature.

This normalization is essential in CL to measure cosine similarity effectively. The combination of these operational steps enhances representation learning by making the obtained features both discriminative and appropriate for usage in CL operations.

### Variational autoencoder

**Fig. 8:**
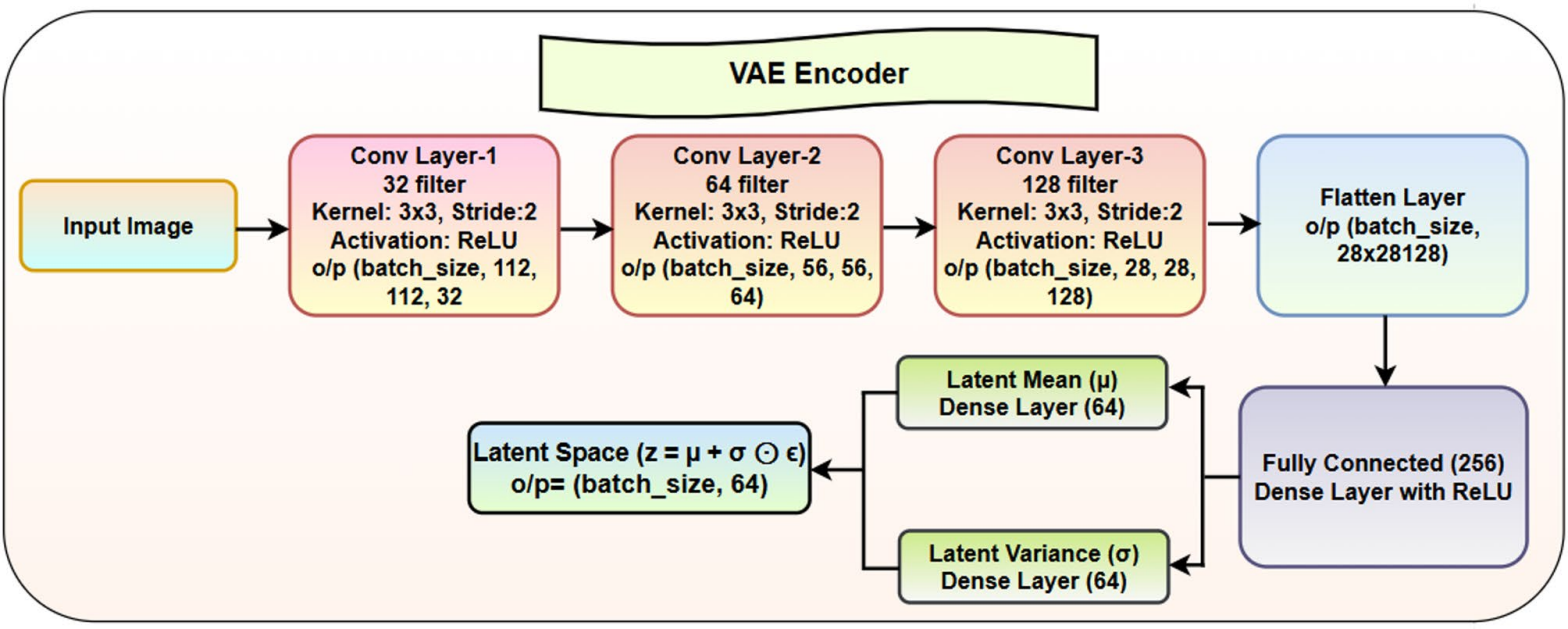
VAE encoder architecture.

VAE is critical component of the proposed model. The encoder’s task is to compress the input image into a smaller latent space, represented by a mean (µ) and variance (σ), from which we can later sample to reconstruct the image using the decoder. The VAE architecture is shown in Fig. 8. Here the encoder performs image compression through its architecture to transform input pictures into latent representations for future image creation. The input image starts a sequential process of three convolutional layers that extract important characteristics from the image data. The initial convolutional layer sets 32 filters alongside a 3 × 3 kernel size and applies a stride value of 2 before the next layers with 64 filters and the final layer containing 128 filters. The non-linear patterns in data become observable when each convolutional layer uses the ReLU activation function during modeling. The stored data at each layer goes through spatial size reduction until it becomes one-dimensional for further processing. After passing through the three convolutional layers, the output is a 4D tensor with shape (batch_size, 28, 28, 128). Before feeding this data into the fully connected (dense) layers, the tensor is flattened into a 1D vector. The flattening process reshapes the data into a vector, which is essentially a long list of all the features extracted by the convolutional layers. Two dense layers receive the flattened vector to calculate latent mean (µ) and latent variance (σ) establishing the probabilistic distribution for latent space. The network learns these two values that define the distribution, which enables it to generate reconstructions through sampling. The latent mean is the center of the latent space distribution, while latent variance defines the spread of the distribution. These two values allow the model to learn a probabilistic input representation.

The formula for calculating the latent mean and latent variance is:

14 $$\:{\upmu\:}=\mathrm{D}\mathrm{e}\mathrm{n}\mathrm{s}\mathrm{e}\:\mathrm{L}\mathrm{a}\mathrm{y}\mathrm{e}\mathrm{r}\:\left(64\right)$$

15 $$\:{\sigma\:}^{2}=\mathrm{D}\mathrm{e}\mathrm{n}\mathrm{s}\mathrm{e}\:\mathrm{L}\mathrm{a}\mathrm{y}\mathrm{e}\mathrm{r}\:\left(64\right)$$

Here, both the mean and the variance are computed using dense layers, where each dense layer outputs a vector of size 64. This ensures that both µ and σ are learned from the data. The sampling process in the latent space relies on the reparameterization trick to generate a latent variable (z). It is given by:

16 $$\:z=\mu\:+\sigma\:\odot\: \epsilon$$

Here, ε∼N(0, I) = Random noise sampled from a normal distribution and ⊙ represents element-wise multiplication. The Kullback-Leibler (KL) Divergence Loss in VAE is a regularization term that ensures the latent space follows a standard normal distribution N(0, I), which is essential for meaningful interpolation and controlled generation of new data samples. Unlike standard autoencoders, which map inputs to fixed latent representations, VAEs learn a probabilistic latent distribution by modeling each latent variable as a Gaussian distribution with mean µ and variance.

The KL divergence loss is given by:

17 $$\:{L}_{KL}=\:-\frac{1}{2}\:\sum\:(1+log{\sigma\:}^{2}-{\mu\:}^{2}-{\sigma\:}^{2})$$

Here log$\:{\sigma\:}^{2}$ ensures numerical stability and helps in computing the divergence, terms $\:{\mu\:}^{2}\:and\:{\sigma\:}^{2}$ measure the deviation of the learned distribution from the unit Gaussian N(0,1).

The data sampling operation allows the model to develop new information and still support learning through backpropagation. A fully connected layer containing 256 units together with ReLU activation shapes the latent variable before it proceeds to the decoder component. The complete architecture lets the VAE encoder develop a probabilistic compact image description from which the model can create new pictures by drawing samples.

**Fig. 9:**
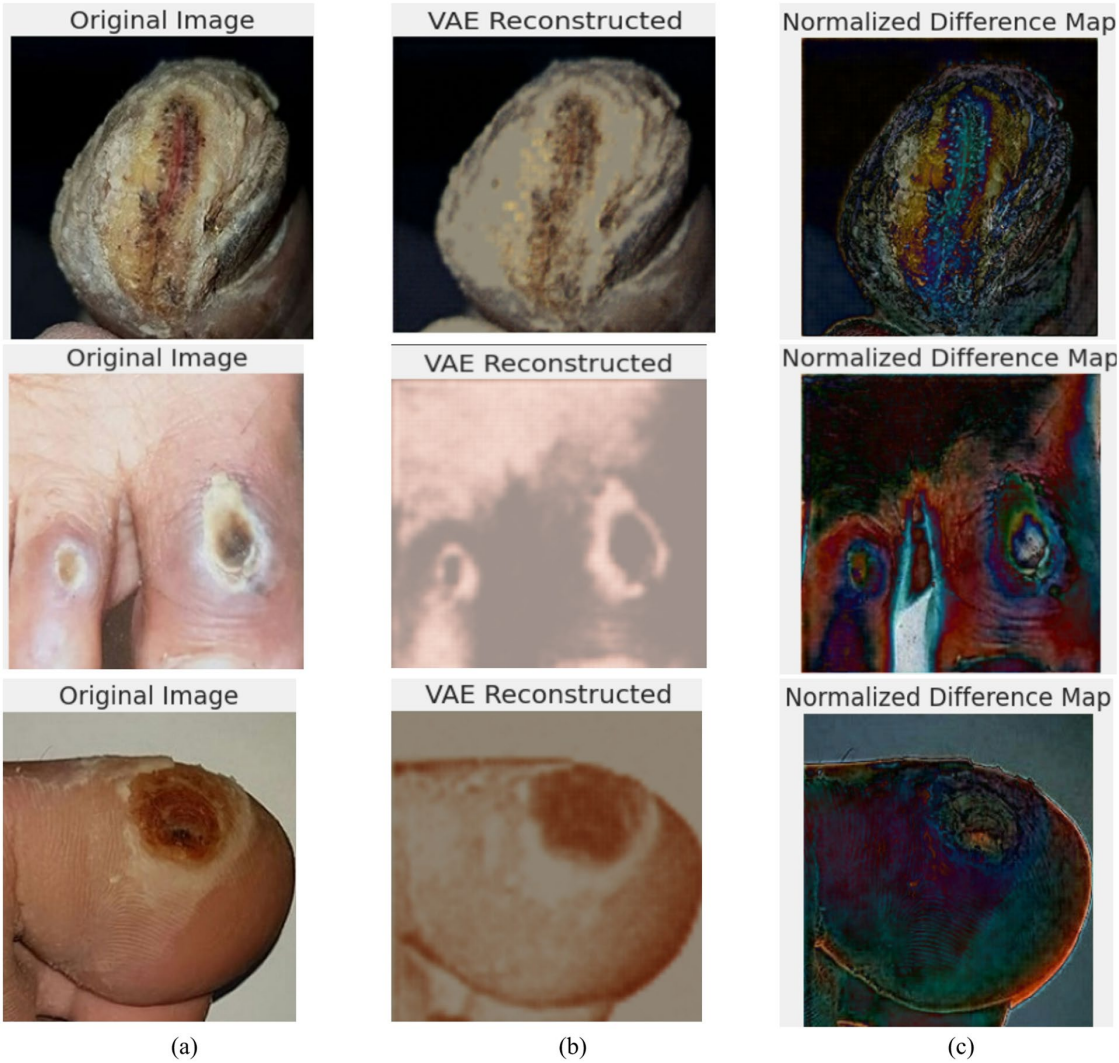
Sample images for VAE-based reconstruction and difference mapping for DFU detection (**a**) original image (**b**) VAE reconstructed (**c**) normalized difference map.

Figure 9 showcases the role of VAE in reconstructing foot ulcer images and generating a normalized difference map for anomaly detection. The leftmost image represents the original DFU image, showing detailed skin texture and ulcer regions. The middle image is the VAE-reconstructed version, where the model attempts to regenerate the input based on learned latent representations. By nature of its unsupervised learning, VAE typically reconstructs the more salient or general features while missing or obscuring subtle anomalies. The far-right image is the normalized difference map that highlights the differences between the original and reconstructed images. It serves to visually enhance these distinctions, highlighting where the model struggled to recreate localized anomalies, thereby helping to underscore possible pathological areas.

These visualization supports unsupervised infected area localization and aids in enhancing model explainability for medical diagnostics.

### VAE decoder

**Fig. 10:**
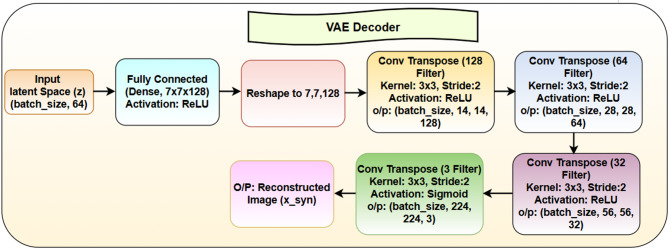
VAE decoder architecture.

The VAE decoder shown in Fig. 10 generates image predictions from latent space representation vector (z) that the encoder has learned throughout training. At the beginning of the process, the input latent space with size (batch_size, 64) receives a fully connected dense layer operation. The 3D feature map with size 7 × 7 × 128 emerges from the layer expansion and representation reshaping to serve as the basis for upsampling. The decoder uses deconvolution layers, also known as transposed convolutional layers, to reconstruct the image while restoring its spatial resolution step by step. The first transposed convolution layer contains 128 filters that function with a 3 × 3 kernel size, stride of 2 pixels and ReLU activation and produces an output of (batch_size, 14, 14, 128). The feature map expands to (batch_size, 28, 28, 64) after another layer with 64 filters employs a 3 × 3 kernel with ReLU activation. The second layer of upsampling applies 32 filters, which produces (batch_size, 56, 56, 32) output shape. The final transposed convolution layer applies 3 filters for RGB channels while using a 3 × 3 kernel, stride of 2 and includes a Sigmoid activation function for image reconstruction normalization between 0 and 1. This operation generates the final reconstructed image. The decoder functions as a generative model that operates in the image domain while mapping between the latent space and image domain for probability distribution learning. Optimizing the quality of generated images requires a reconstruction loss that determines the difference between original images and reconstructed images using typical binary cross-entropy or mean squared error. The KL divergence loss preserves the accuracy in the latent space by keeping the learned distribution close to the standard normal distribution, avoiding overfitting and enabling smooth interpolation in the latent space. The VAE decoder generates high-quality realistic images with meaningful generated latent representations using a specified method.

The mathematical expressions used during the VAE decoding functions are stated below:

## Decoder transformation function

The decoder learns a mapping function pθ(x∣z) to reconstruct the input image x from z:

18 $$\:{x}_{syn}={f}_{decoder}\left(z\right)$$

- Loss Function: The Reconstruction Loss (Binary Cross-Entropy) ensures that the generated image $\:{x}_{syn}$​ is close to the original x and is given by:

19 $$\:{L}_{reconst}=\:\sum\:x\:log\left({x}_{syn}\right)+(1-x)\mathrm{l}\mathrm{o}\mathrm{g}(1-{x}_{syn})$$

The KL Divergence Loss (from the encoder) regularizes the latent space. Total VAE Loss is given by:

20 $$\:{L}_{VAE}=\:{L}_{reconst}+\beta\:{L}_{KL}\:$$

where β is the hyperparameter that controls the trade-off between the quality of reconstruction and the regularization of the latent space. In summary, every layer in the VAE decoder increases the spatial structure, gradually increasing the resolution until it matches the size of the original input. The last layer consists of a Sigmoid activation function, normalizing pixel values in the range from 0 to 1, hence guaranteeing the result will be a valid image. The VAE is trained through a combination of Reconstruction Loss-the assessment of how well the generated image fits the original-and KL Divergence Loss-maintaining a structured and continuous latent space. This dual-goal training enables the VAE to generate realistic images while keeping a coherent and meaningful latent representation.

## Results and discussion

This section presents a detailed analysis of the experimental results and evaluates the effectiveness of the proposed model.

### Experimental setup

N experimental setup was designed to detect conditions of DFU by differentiating between healthy and infected skin areas using an improved DenseVAE-CL model leveraging transfer learning with a pre-trained DenseNet121 backbone. The model was updated to extract DFU-specific visual characteristics from the accessible Kaggle DFU dataset, which contains 2,673 images. The data was split into training, validation, and testing sets following an 80:10:10 ratio, which provided a balanced representation for both learning and assessment. To improve generalization, a thorough data augmentation approach was utilized, involving horizontal and vertical flips, random rotations (± 20°), width and height translations (± 0.2), a zoom range of 0.2, and intensity normalization to [0,1]. All experiments were performed with a constant random seed of 123 to ensure reproducibility. The Adam optimizer was employed for model optimization, with a starting learning rate of 0.001, momentum set at 0.8, and weight decay regularization of 1e-4 to avoid overfitting. The batch size was configured to 32, and the training lasted for 60 epochs. The classification utilized the cross-entropy loss function, whereas the representation learning phase was directed by contrastive loss. The VAE sub-module was trained in conjunction with the classification network to improve the reconstruction of latent features. This setup offered a reliable and computationally efficient training process, resulting in steady convergence and peak accuracy during five-fold validation. The model was executed in a TensorFlow 2.12 environment using an NVIDIA Tesla T4 GPU (16 GB VRAM).

### Results of Densenet121

**Fig. 11:**
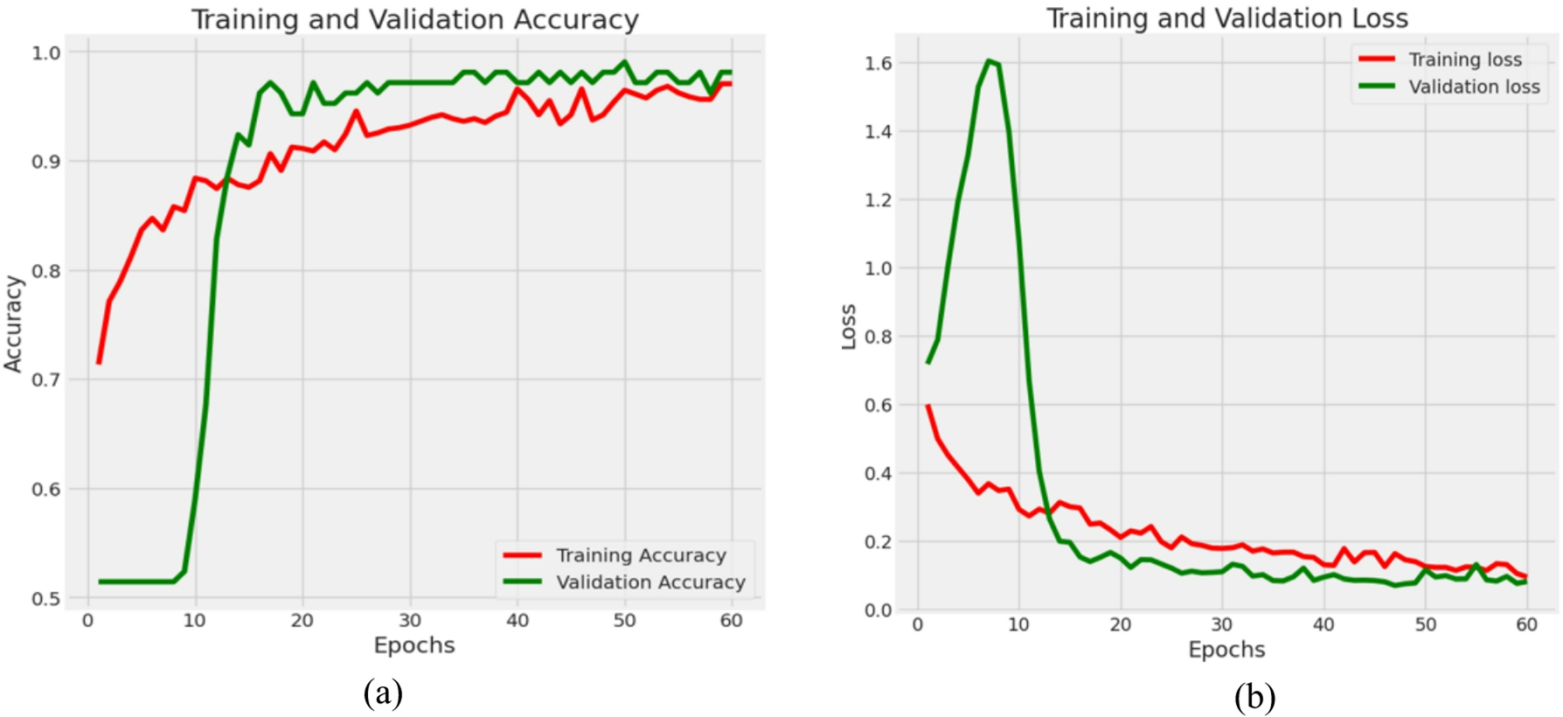
Progression of training and validation metrics for DenseNet121 (**a**) Accuracy (**b**) Loss.

This section presents the results for DenseNet121. Figure 11 represents in detail the training and validation results over 60 epochs, including both loss and accuracy metrics with respect to model convergence and generalization ability. Figure 11(a) reflects this positive trend. The training accuracy increases rapidly from approximately 0.65 to more than 0.95 toward the end of training. Similarly, the validation accuracy starts somewhat higher compared to the training accuracy and increases linearly, finally stabilizing around 0.965. This consistent increase in training and validation accuracy shows that the model learns the important features. The close tracking of training accuracy with validation accuracy over epochs supports the concept of a well-generalized model with low overfitting.

In Fig. 11b, loss values for training and validation sets decrease steadily with increasing number of epochs. At the beginning, the training loss is about 0.72, decreasing drastically in the first few epochs, which means it can learn fast. The validation loss follows a similar trend, though with slight variation in the first few epochs, probably because the model is tuning its parameters and the natural variability in the validation data. After epoch 7, both curves stabilize and slowly decrease until finally obtaining values below 0.1 at epoch 20. In general, from these visualizations, it can be seen that the model learns well, as evidenced by a decrease in loss and an increase in accuracy on both training and validation sets. There is good consistency and proximity of validation metrics to those of training across all metrics, which signals a good model architecture, proper training, and a well-preprocessed dataset.

Table 1 presents the metrics for the DenseNet121 model. Assessment metrics show how well the classification model distinguished between Abnormal (Ulcer) and Normal categories. The model achieved a precision of 0.98 for the Abnormal (Ulcer) class, which means that 98% of the cases identified as ulcers were correct, with very few false positives. The model shows a recall of 0.94, which means it correctly identified 94% of true ulcer cases while having few missed cases. The general performance for recognizing this class is a strong F1-score of 0.96, combining precision and recall metrics. The Normal class yielded a precision rate of 0.97, including a recall rate of 0.96, since the model identified most actual normal cases and the misclassification of ulcers as normal was very minimal. The Normal class F1-score is 0.965, reflecting consistent results in classification. It also obtained an accuracy of 0.969 (96.9%) upon application to classify all test samples totaling 534; this means, indeed, that most of the instances observed were effectively identified. These assessment metrics thus reveal the model’s effective and reliable ability in correctly identifying both ulcer and normal cases.

**Table 1:** Performance measures for base Densenet121.

Class	Precision	Recall	F-1	Accuracy
Abnormal (Ulcer)	0.98	0.94	0.96	0.969
Normal	0.97	0.96	0.965	

**Fig. 12:**
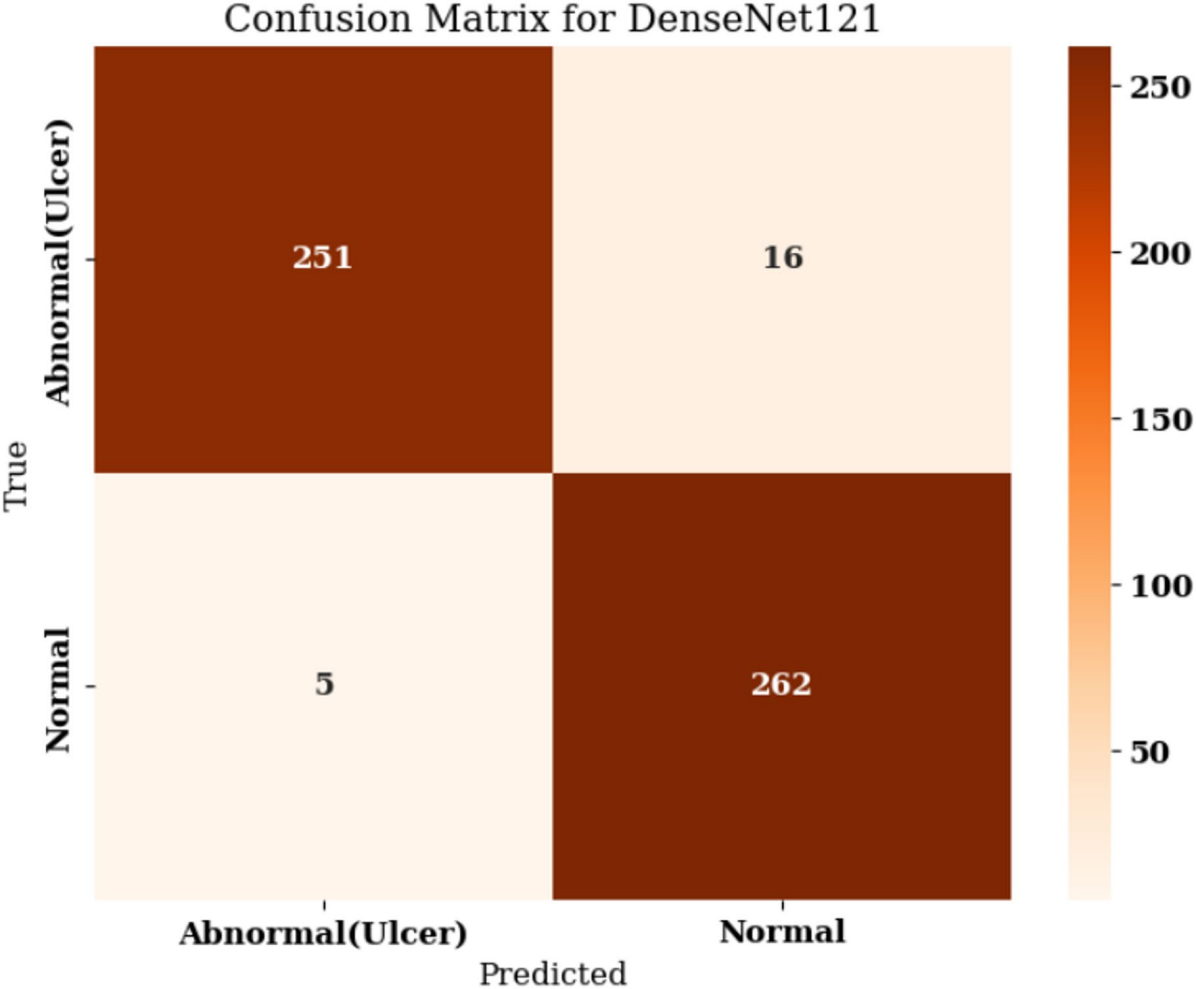
Confusion matrix for base DenseNet121.

The confusion matrix shown for the DenseNet121 model in Fig. 12 offers a clear summary of the classification performance regarding the two categories: Normal and Abnormal (Ulcer).

Out of the total 534 test samples, the model correctly identified 251 Abnormal (Ulcer) cases and 262 Normal cases, reflecting very good accuracy. It misclassified 16 actual ulcer cases as Normal (false negatives) and wrongly identified 5 Normal cases as Abnormal (false positives). That means this model is highly precise for the Abnormal class; it rarely misses classifying an ulcer case and has high recall, thus correctly detecting most actual ulcers. The small number of misclassifications shows the model’s robustness and reliability in distinguishing between the two classes, which is also reflected in the overall accuracy of approximately 96.9%. The DenseNet121 model thus performs effectively in clinical image classification tasks involving foot ulcers. From the confusion matrix, the model attains a Sensitivity of 0.940 and a Specificity of 0.981, indicating strong detection of ulcer cases and very low false-positive rates for normal cases.

**Fig. 13:**
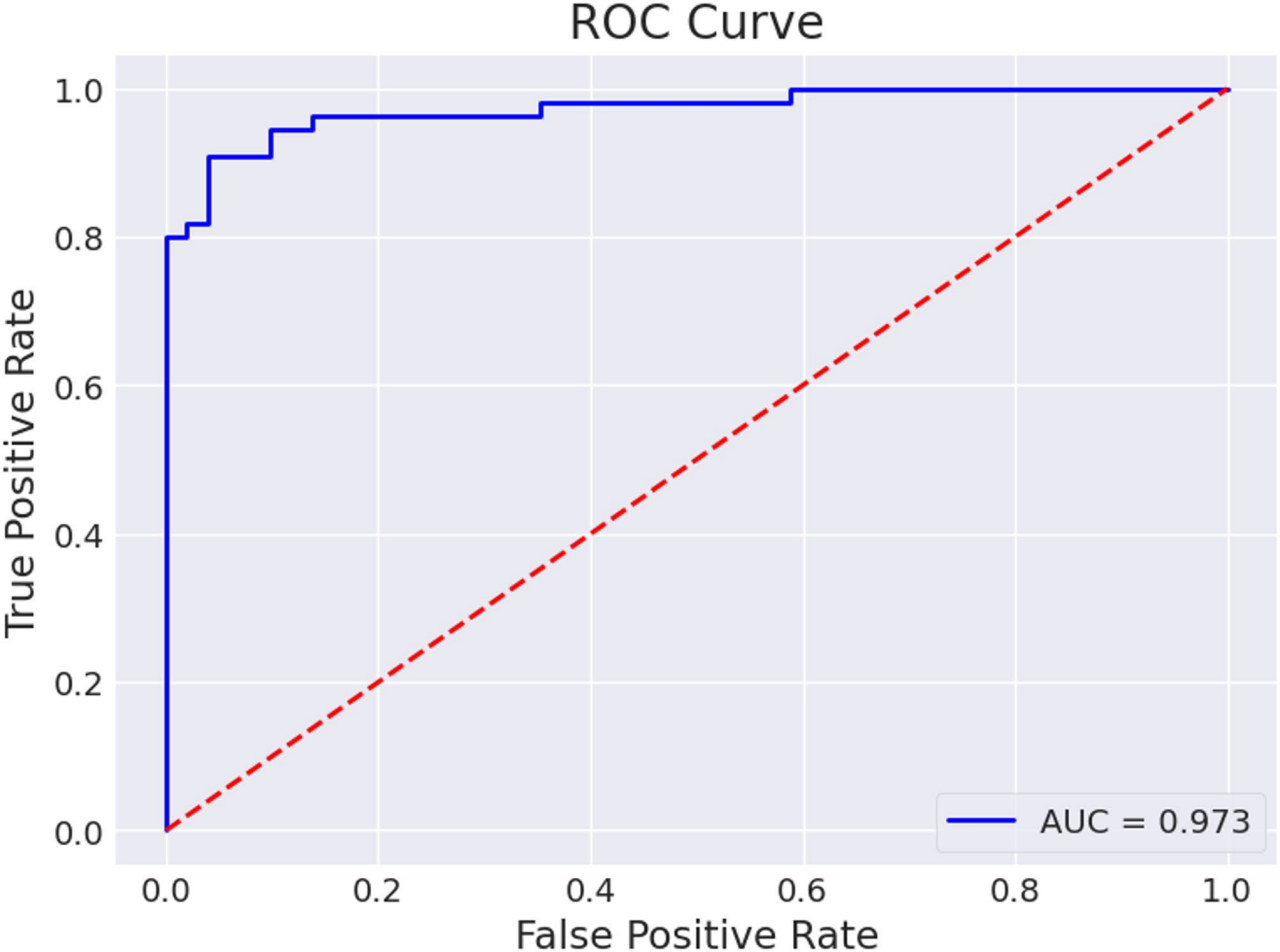
ROC Curve for base DenseNet121 model.

Figure 13 displays the ROC curve for base model, from which it can be seen that the curve shows an AUC of 0.973, indicating strong but slightly lower discriminative performance compared to the VAE-augmented and full DenseVAE-CL models. While the model effectively distinguishes ulcer and non-ulcer cases, the relatively lower AUC suggests that training only on real images limits its ability to capture the full variability of lesion characteristics.

### Results of DenseNet121 with contrastive learning

**Fig. 14:**
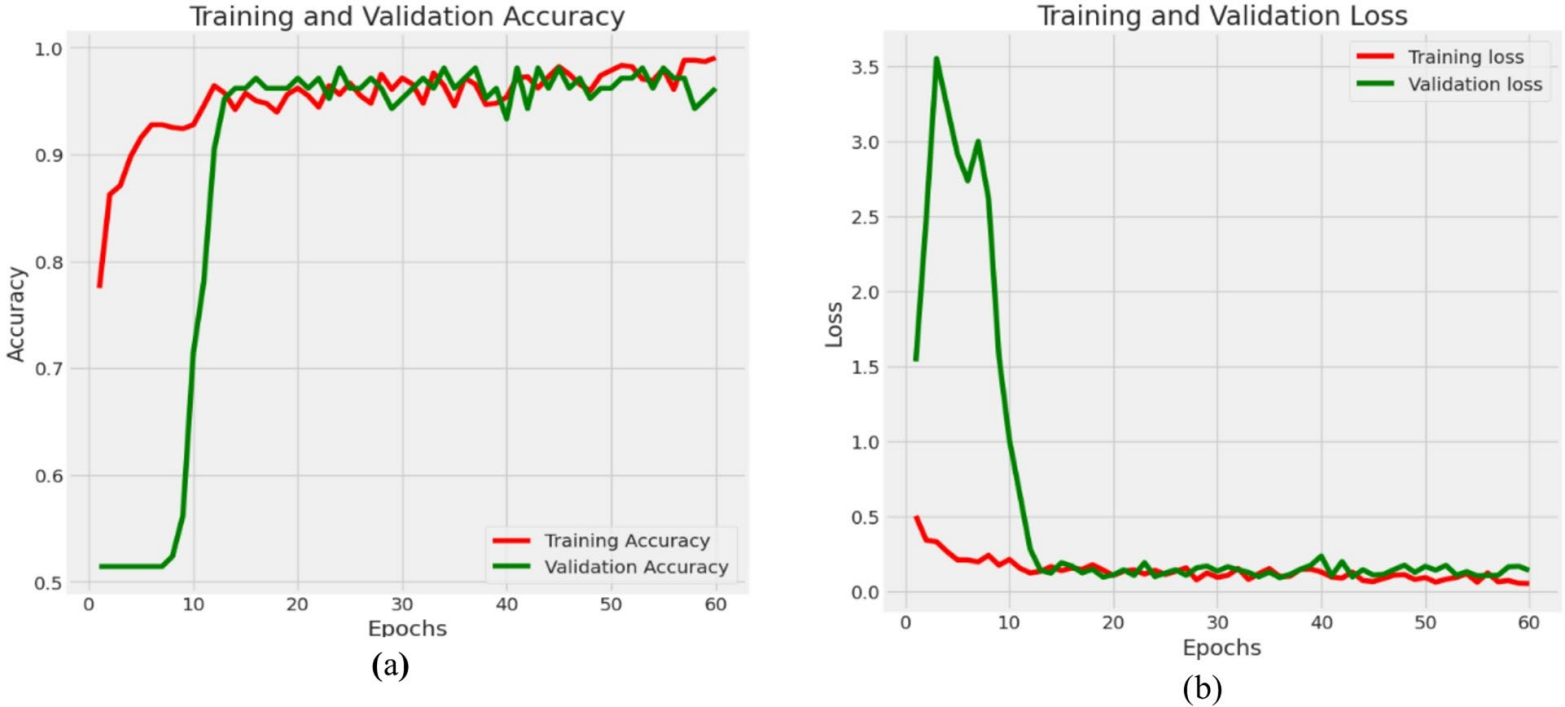
Progression of training and validation metrics for DenseNet121 with CL (**a**) Accuracy (**b**) Loss.

The provided graphs of Fig. 14 illustrate the training and validation performance of the DenseNet121 with CL model over 20 epochs, concerning accuracy and loss. The first graph shows the progression of training and validation accuracy, while the second graph presents the corresponding training and validation loss curves.

In the accuracy plot of Fig. 14a, both training and validation accuracy exhibit a consistent upward trend across epochs. The training accuracy begins at 0.55 during the first iterations before achieving complete accuracy of 1.0 at the 20th epoch. The validation accuracy demonstrates an almost equivalent pattern by starting at 0.6 before reaching 0.97 at the last epoch mark. The model demonstrates excellent performance in learning efficiently because the two curves remain close to one another throughout all training cycles while avoiding substantial overfitting. The training and validation loss decreases steadily as indicated in Fig. 14(b) while accuracy increases progressively. Both training loss and validation loss begin above 1.0 and become nearly 0.05 while validation loss decreases in parallel to reach approximately 0.06 at the end of training. The parallel decline of training and validation loss lines proves the model achieves effective learning while maintaining data unpredictability.

Overall, these graphs demonstrate that the DenseNet121 model has achieved high accuracy with low loss for both training and validation sets. This implies an excellent fit, generalization, and minimal overfitting, making it a highly effective architecture for the given classification task involving medical image data.

Table 2 describes the performance measures for DenseNet121 with CL. Analysis of Table 2 demonstrates that the model produced precision results of 0.99 for the Abnormal (Ulcer) category where all predictions for ulcers turned out to be valid with just a tiny number of incorrect classifications. This model recalled 0.97 of the true ulcer cases thus successfully detecting 97% of actual ulcers while failing to identify a minimal number. The mathematical measure of F1-score at 0.98 proves that the model optimally balances recall and precision during ulcer detection tasks. The model achieved significant scores for the Normal class with a precision rate of 0.97 alongside recall of 0.98 and F1-score of 0.97.

**Table 2:** Performance measures for Densenet121 with CL.

Class	Precision	Recall	F-1	Accuracy
Abnormal (Ulcer)	0.99	0.97	0.98	0.975
Normal	0.97	0.98	0.97	

**Fig. 15:**
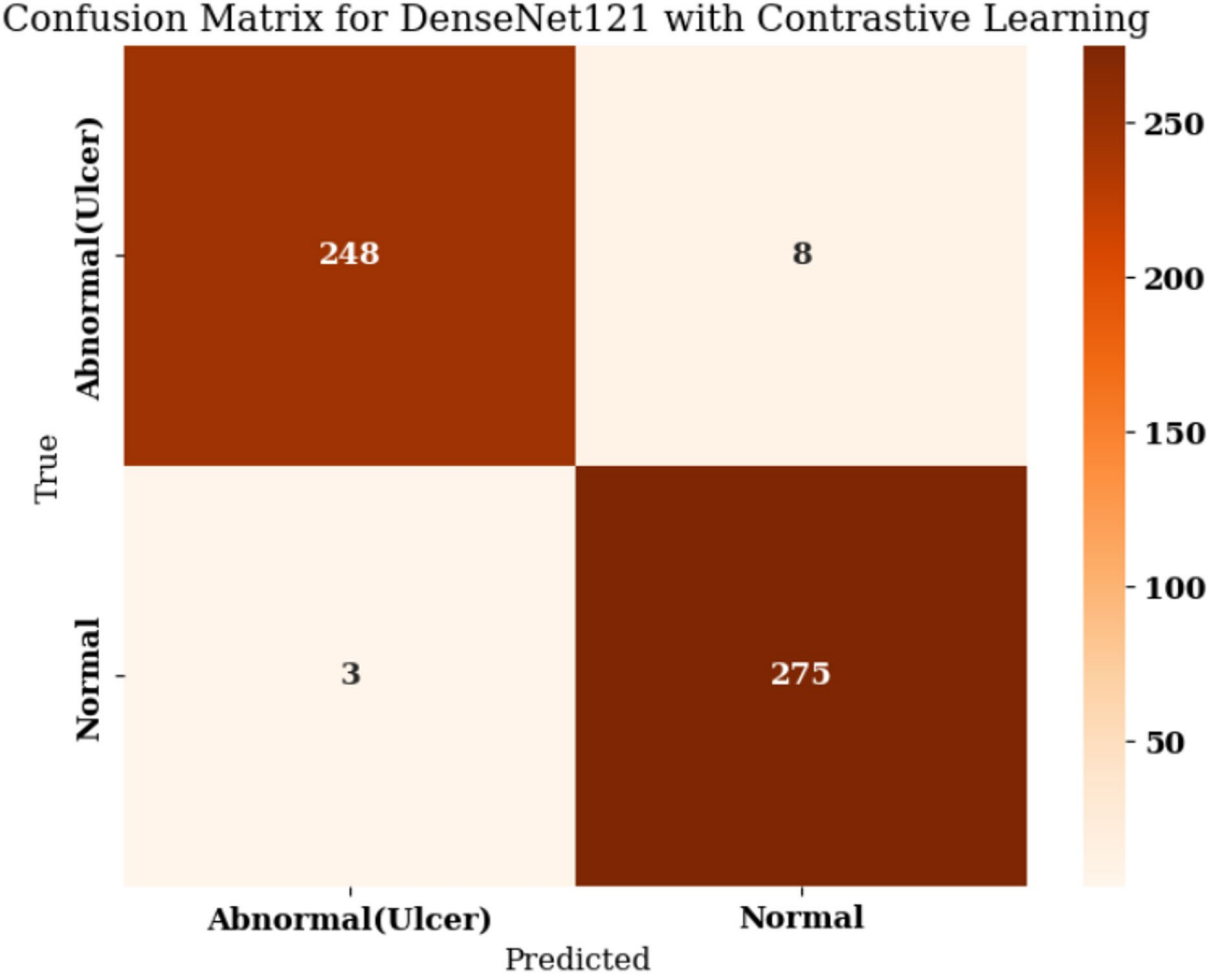
Confusion matrix for Densenet121 with CL.

It finds most of the instances of healthy skin while generating few errors, which confirms that the model has excellent diagnostic accuracy. The model presents 97.5% accuracy in overall task performance while ensuring consistent functionality for both ulcerated and normal foot tissues. Experimental data demonstrates that this model proves to be a suitable solution in practical medical image evaluation tasks that aim to find foot ulcers in a fast and accurate way. Out of 256 real Ulcer cases, the model correctly predicted 248 of them (true positives) but has wrongly grouped eight cases as Normal instances (false negatives), as portrayed in Fig. 15. The model is very sensitive in finding ulcers, hence giving a recall performance of about 0.97 for the ulcer category. In the case of a Normal class, it has classified 275 out of 278 samples correctly (true negatives) while misinterpreting 3 as Ulcer (false positives), yielding a precision of approximately 0.99 regarding ulcer predictions.

This confusion matrix represents extremely high model performance, as it shows a high precision and a high recall for both classes. The low number of misclassifications here supports the success of incorporating CL into the proposed approach, which enhances its capability of distinguishing between visually similar yet clinically important features and hence generalizing better to unseen test data. Using the given counts (TP = 248, FN = 8; TN = 275, FP = 3), sensitivity amounts to 0.969 while specificity reaches 0.989, thus proving that CL enhances both true-positive identification and true-negative filtering.

**Fig. 16:**
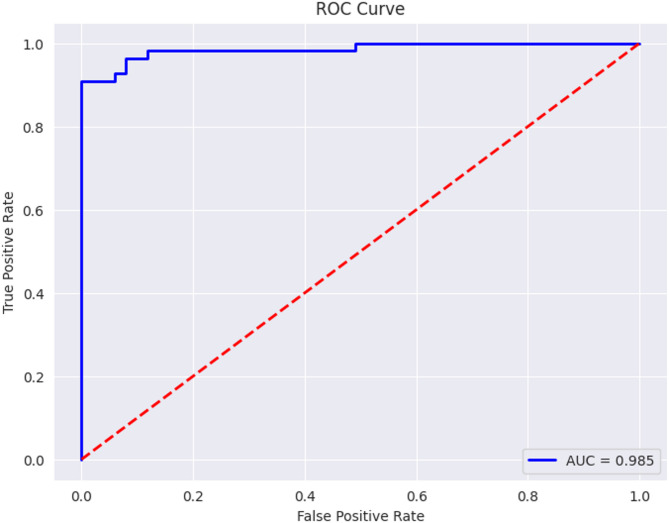
ROC curve for Densenet121 with CL.

Figure 16 shows ROC curve for Densenet121 using CL. The AUC score is 0.985, which shows that though the baseline DenseNet121 has a promising capability in differentiating ulcer and non-ulcer images, it still can improve, and this improvement is later achieved by the proposed DenseVAE-CL model.

### Results of proposed DenseVAE-CL (Densene121 with contrastive learning and VAE Autoencoder)

**Fig. 17:**
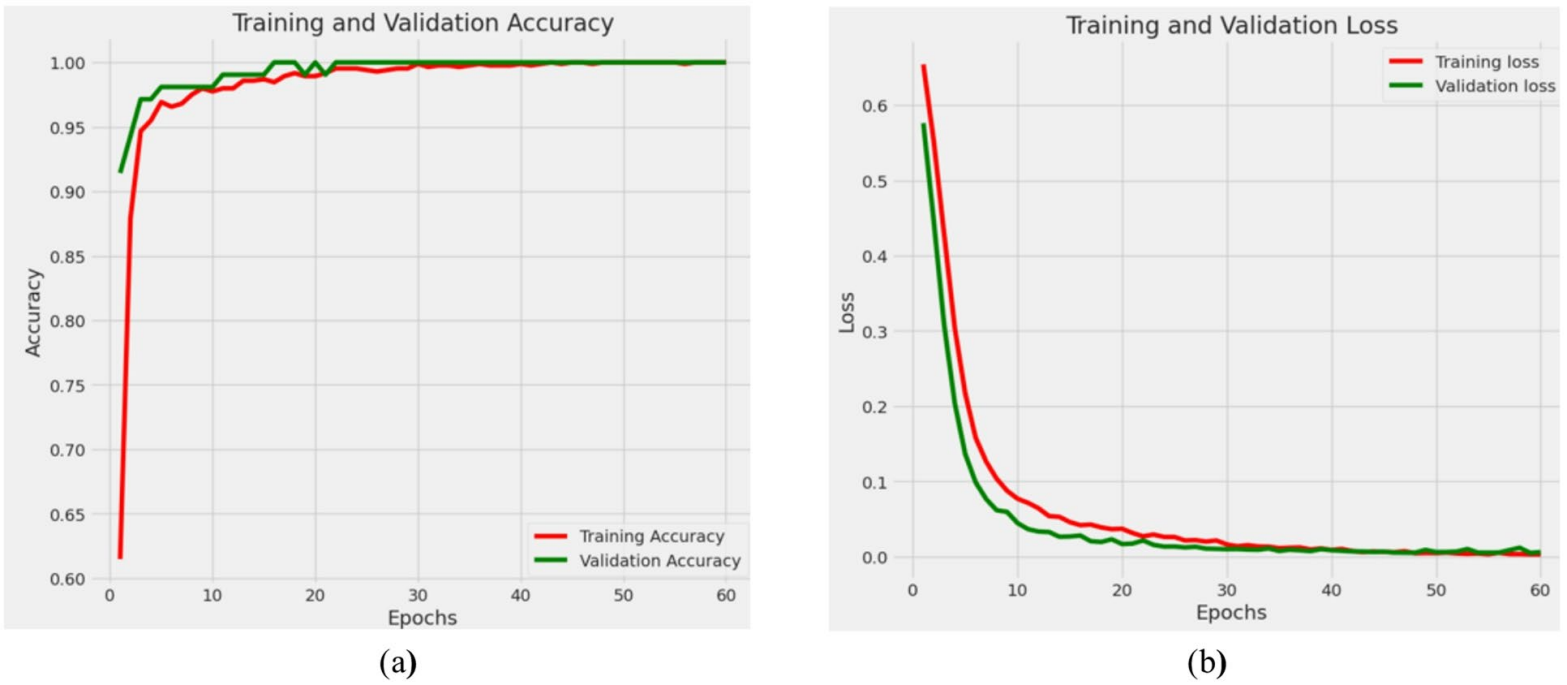
Progression of training and validation metrics for proposed DenseVAE-CL model (**a**) Accuracy (**b**) Loss.

This section presents the results of the performance of the proposed DenseVAE-CL model. Graphs in Fig. 17 demonstrate the training vs. validation performance of the DenseNet121 model using CL and VAE Autoencoder, focusing on accuracy and loss trends.

In the accuracy plot Fig. 17a, the training accuracy stays steadily high during the training process, beginning over 85% and swiftly approaching almost 100% by the third epoch, where it levels off for the rest of the training. Conversely, the validation accuracy exhibits considerable variation in the early epochs, fluctuating between 70% and 90%, likely due to the model adapting to intricate validation data or restricted initial generalization. Nevertheless, following the eighth epoch, the validation accuracy shows a significant increase and stabilizes, closely matching the training accuracy and achieving about 98–99% in the last epochs. This convergence shows that the model effectively learned to generalize on new data following the initial fluctuations. The loss graph illustrated in Fig. 17b reinforces this finding. The training loss steadily declines and levels out at a significantly low value, demonstrating that the model matches the training data exceptionally well. The validation loss, on the other hand, initially varies significantly, hitting highs over 1.6 during the 3rd to 5th epochs. Despite this, following the seventh epoch, the validation loss consistently decreases and aligns with the training loss, indicating enhanced model generalization and diminished overfitting. In general, these graphs indicate that the model attained excellent accuracy and minimal loss across both datasets, signifying robust and reliable performance. The application of CL probably aided the model in acquiring detailed feature representations, allowing it to effectively differentiate subtle differences between normal and abnormal skin conditions.

Table 3 presents the classification report, which is a testament to the brilliant performance for a binary classification system between abnormal (Ulcer) and normal skin conditions. For the Abnormal (Ulcer) class, the precision turned out to be perfect, 1.0, showcasing that every ulcer predicted was correct with no false positive results. The model correctly identified 99% of the existing ulcer cases in the dataset, demonstrating an incredibly low false-negative rate. The F1-score of 0.995 is indicative of excellent balance between precision and recall, acting as a metric on model reliability in diagnosing ulcers. The Normal class incorporates similar results compared to the Ulcer class, with a precision of 0.99 and a recall of 1.0. The actual Normal cases were entirely detected by the model which produced minimal incorrect positive results. The F1-score value of 0.995 demonstrates that the model keeps an exceptionally high level of accuracy when detecting normal skin conditions. Extremely low misclassification rates combined with a 0.99 overall accuracy rate demonstrate that the model shows excellent performance in predicting unseen data with great robustness. The diagnostic accuracy achieved by the model proves useful in medical applications because it can prevent both erroneous alarm signals and diagnostic errors.

**Table 3:** Performance measures for the proposed DenseVAE-CL Model.

Class	Precision	Recall	F-1	Accuracy
Abnormal (Ulcer)	1.0	0.99	0.995	0.996
Normal	0.99	1.0	0.995	

**Fig. 18:**
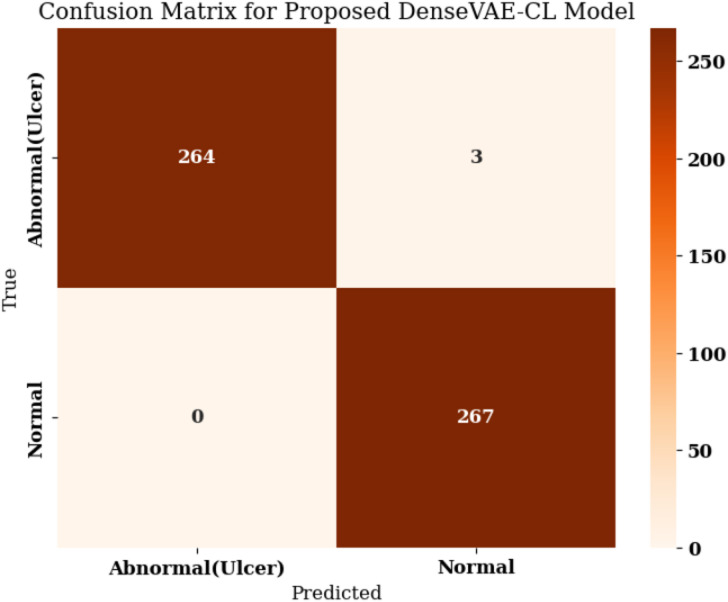
Confusion matrix for the proposed DenseVAE-CL Model.

The confusion matrix shown in Fig. 18 represents the classification performance of the Proposed DenseVAE-CL Model for detecting Abnormal (Ulcer) and Normal skin conditions on a test set of 534 images. The matrix indicates a highly accurate model with excellent generalization capabilities. Out of 267 actual Abnormal (Ulcer) cases, the model correctly predicted 264 as ulcer (true positives) and misclassified only 3 as normal (false negatives). This corresponds to a recall of approximately 0.99 for the ulcer class, showing the model’s effectiveness in correctly identifying nearly all positive cases.

Also, the precision is 1.0 since there are no false positives in the ulcer category, which suggests that every case the model identified as an ulcer was actually an ulcer case. In the Normal class, all 267 were classified correctly, giving a recall of 1.0, with only three false positives due to incorrectly classified ulcer cases. This results in an approximate precision of 0.99 for the normal class. Finally, with TP = 264, FN = 3, TN = 267, FP = 0, the proposed model obtained a sensitivity of 0.989 and a specificity of 1.000, giving a perfect separation between ulcer and normal cases. Another way to look at this performance is represented by the confusion matrix, indicating that 531 out of 534 subjects were correctly predicted, which sets an accuracy of about 99%. This means that DenseVAE-CL demonstrates strong performance on publicly available DFU image data and shows promise for future real-world applications following external validation.

**Fig. 19:**
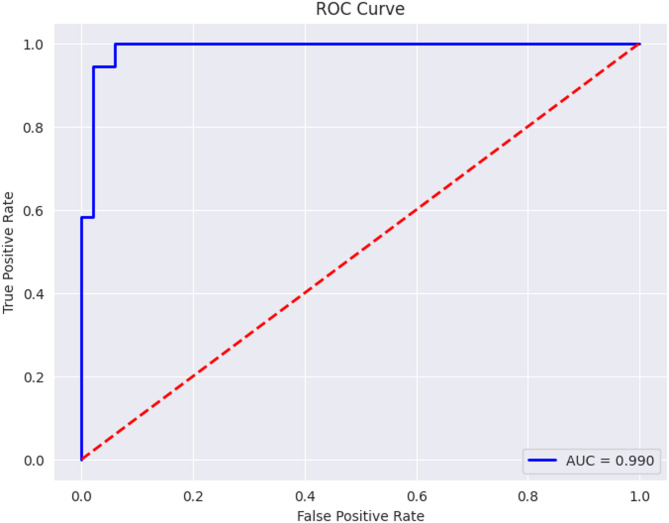
ROC curve for proposed DenseVAE-CL Model.

The ROC curve of the proposed DenseVAE-CL model is shown in Fig. 19. This ROC curve depicts how the proposed DenseVAE-CL model is effective with both real images and VAE generated synthetic images. The model achieves an AUC of 0.990 which is a considerable improvement over the baseline DenseNet121 (AUC = 0.973) and the DenseNet121 with contrastive learning (AUC = 0.985). The curve is placed close to the upper-left side which means that the classification of ulcer and non-ulcer categories is almost perfect. This result confirms that VAE-based synthetic augmentation in combination with CL significantly enhances the capability of the model to discriminate features and its capacity to generalize.

### Class wise mean confidence and expected calibration error

**Fig. 20:**
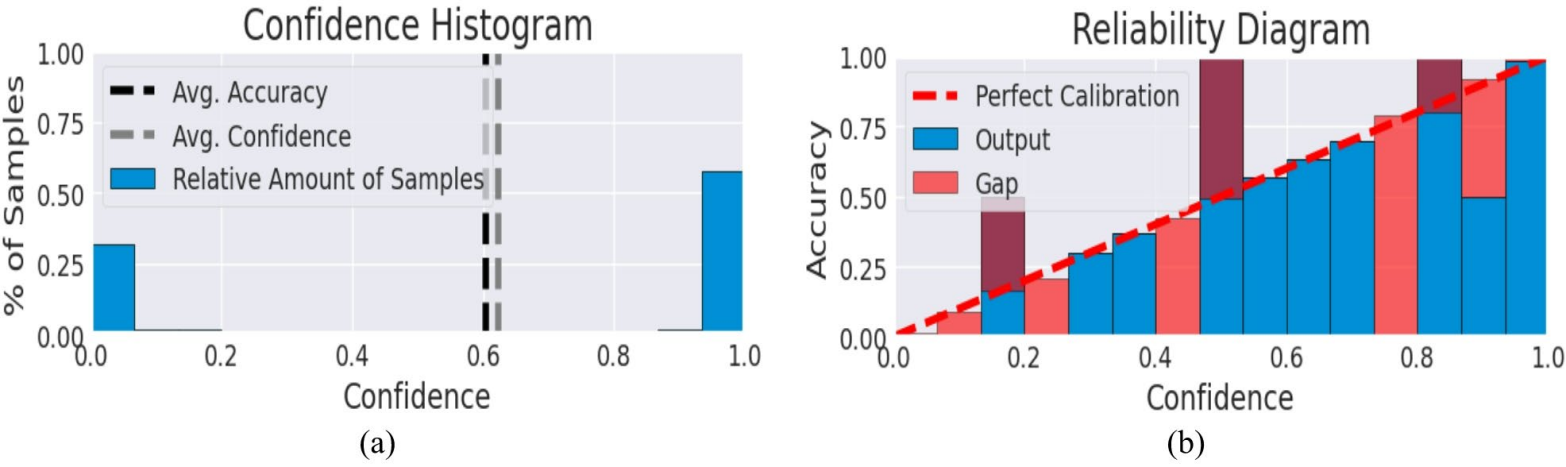
Confidence histogram and reliability diagram for model calibration.

The expected Calibration Error (ECE) and the average confidence per class give data on the reliability of the model and the level of confidence on which it will predict in each of the classes. The mean confidence is a measure of the confidence of the model whenever making predictions in each of the classes, the ECE is an evaluation of the relationship between the model prediction confidence and actual performance. Figure 20 presented the results of the model that give an evaluation of the confidence and calibration of the model depending on a specific class. The mean confidence of the category Abnormal (Ulcer) is 90.31, and that of the category Normal (Healthy skin) is 96.92. This indicates that the model is more confident when trying to predict normal (healthy) skin than when there is an issue of the ulcer. Such difference in confidence is expected in medical imaging where a pathological tissue could be more heterogeneous and difficult to classify compared to normal tissue. Together with confidence, the Expected Calibration Error (ECE) was calculated to compare the predicted probabilities and the actual accuracy. There was an ECE of 0.0457 (or 4.57), meaning a low level of miscalibration, on both categories. This implies that the predicted probabilities of the model are usually within the range of 4.6% of the actual values, meaning that the model usually is well-calibrated.

A lower ECE means that the model confidence is a good predictor of its accuracy, which is very important in sensitive applications like medical diagnosis. Thus, the results demonstrate that not only does the model perform confidently, but it also maintains good calibration across both ulcer and healthy skin classes.

### Ablation study

The comprehensive ablation analysis in Table 4 assesses four variations of the suggested framework to discern the effects of VAE-based augmentation and contrastive learning. The baseline DenseNet121 + SE model, which was trained solely on real augmented images, delivered impressive results (precision 0.981, recall 0.975, accuracy 0.975); however, its reduced sensitivity (0.940) suggests difficulties in identifying subtle ulcer patterns. The use of synthetic images generated by VAE without contrastive learning (DenseNet121 + SE + VAE) enhanced all metrics, achieving a precision of 0.982, recall of 0.980, accuracy of 0.984, and sensitivity of 0.955. This verifies that synthetic augmentation enhances intra-class diversity and aids the model in generalizing more effectively to variations in lesions.

The CL-only model (DenseNet121 + SE + CL) improved discriminative representation learning, reaching a precision of 0.987, recall of 0.984, accuracy of 0.989, specificity of 0.989, and sensitivity of 0.969. These enhancements indicate that contrastive learning enhances class differentiation and decreases misclassification. The complete DenseVAE-CL model, which combines VAE augmentation, contrastive learning, and SE recalibration, attained the best performance on all metrics precision 0.995, recall 0.994, accuracy 0.996, specificity 1.000, and sensitivity 0.989. These findings illustrate the synergistic advantages of VAE and CL: VAE boosts feature variety, whereas CL refines embedding distinction.

**Table 4:** Ablation study analysis.

Model	Training Data	Precision	Recall	F1-Score	Accuracy	Specificity (TNR)	Sensitivity (TPR)	ROC–AUC
DenseNet121 + SE	Real augmented	0.981	0.975	0.978	0.975	0.981	0.940	0.973
DenseNet121 + SE + CL	Real augmented	0.987	0.984	0.986	0.989	0.989	0.969	0.982
DenseNet121 + SE + VAE	Real + Synthetic	0.982	0.980	0.982	0.984	0.983	0.955	0.977
**Proposed DenseVAE-CL**	**Real + Synthetic**	**0.995**	**0.994**	**0.995**	**0.996**	**1.00**	**0.989**	**0.990**

**Fig. 21:**
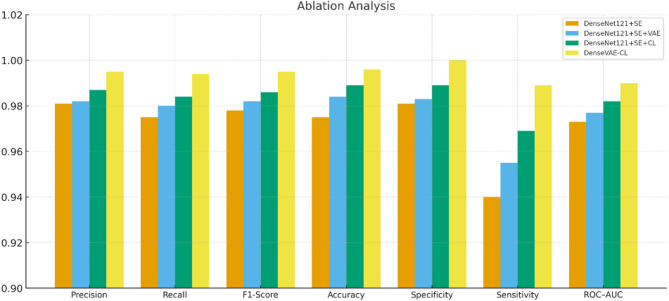
Ablation analysis comparative plot.

Overall, the progression from the baseline to VAE-only, CL-only, and finally the DenseVAE-CL model shows consistent improvements, confirming that combining generative augmentation with contrastive learning yields the most robust and reliable DFU classifier. Figure 21 presents the graph of the performance analysis in the form of the improvement of the base DenseNet121 model due to the addition of CL and VAE Autoencoder.

The chart is a clear display of the gradual improvement in the performance as upgrading of architecture is done. The reference to DenseNet121 + SE provides a strong baseline, but the addition of CL significantly improves the ability of the model to distinguish between classes, leading to high scores in all indicators. The highest improvement is observed in the Proposed DenseVAE-CL model that achieved the highest score in all four measures. This visually shows that VAE combined with contrastive objectives was successful in improving feature representation and discrimination.

### Evaluation of model robustness through 5-fold validation

In order to test the strength and the power of generalization of the proposed DenseVAE-CL framework, the DFU Kaggle data with 2,673 images underwent a five-fold cross-validation procedure. In each of the folds, a total of 80% of the images were used to train, 10% to validate and 10% to test. Table 5 shows the overall performance of the five folds. The model demonstrated exceptionally consistent outcomes, attaining an average Accuracy of 0.994 ± 0.002, Precision of 0.993 ± 0.003, Recall of 0.992 ± 0.003, F1-score of 0.993 ± 0.002, and AUC of 0.996 ± 0.001. All these consistent outcomes among folds demonstrate the reliability of the proposed approach and confirm the fact that generative learning using VAE and a contrastive representation learning can enhance the resilience of the model even with a limited dataset. This stability is also indicative of the fact that the model is effective in removing overfitting and determining important features that are required to classify DFU accurately.

**Table 5:** Fold cross validation results.

Fold	Accuracy	Precision	Recall	F-1	ROC-AUC
Fold 1	0.992	0.991	0.990	0.991	0.995
Fold 2	0.995	0.994	0.993	0.993	0.994
Fold 3	0.983	0.981	0.983	0.982	0.989
Fold 4	0.994	0.993	0.992	0.993	0.995
Fold 5	0.994	0.992	0.990	0.991	0.992
Mean ± SD	0.992 ± 0.005	0.990 ± 0.005	0.991 ± 0.006	0.990 ± 0.004	0.993 ± 0.003

### Role of the VAE module in feature learning and anomaly localization

**Fig. 22:**
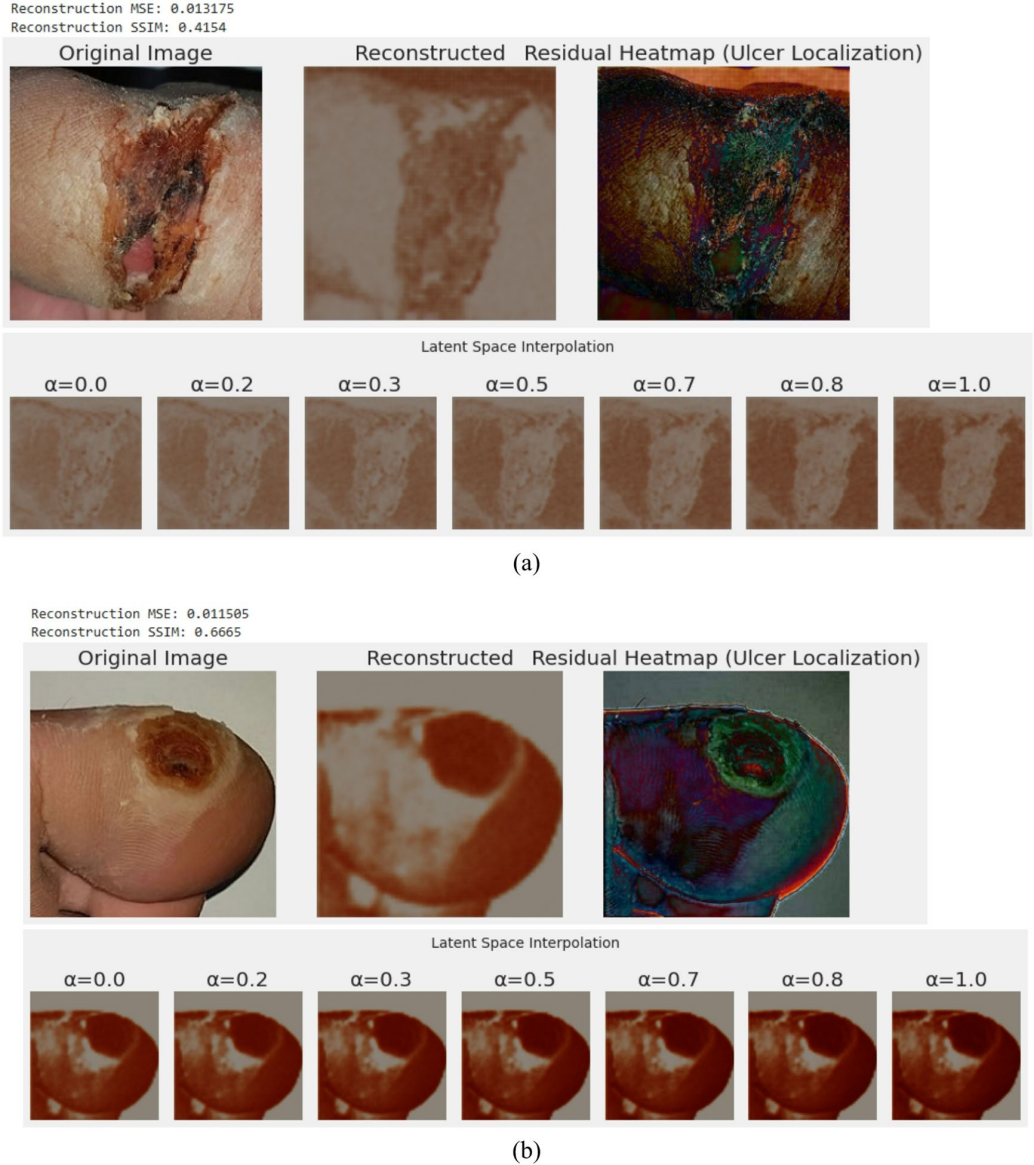
VAE-based reconstruction and latent representation of DFU samples for feature learning and anomaly localization for (**a**) Severe ulcer case with rough surface and irregular wound (**b**) Localized ulcer with defined circular lesion.

In an attempt to improve the arguments behind the inclusion of the VAE element, a latent space analysis and extensive reconstruction was performed. Figure 22 presents the representative examples of the original DFU images, their reconstructions with VAE, and the respective residual heatmaps. The reconstruction error, which is represented as pixel-wise differences, highlights the lesion areas with a high reconstruction loss, thereby highlighting the capacity of the model to estimate the edges of ulcer and irregular surfaces. The VAE Reconstruction and Residual Heatmap of two sample images are depicted in Fig. 22. The initial ulcer image, its reconstruction with the help of VAE, and the residual heatmap that highlights the region of the highest reconstruction error in terms of ulcer tissue are shown in Fig. 22a. The VAE can recreate the morphology of the entire wound with the Mean Squared Error (MSE) of 0.013 and Structural Similarity Index Measure (SSIM) of 0.415, whereas the residual map clearly shows where necrotic and inflamed wounds are located, which proves the ability of the network to distinguish between the pathological difference. Latent space interpolation (bottom row) also shows that two encoded representations of ulcers can also shift seamlessly, showing that the acquired latent manifold has continuity in the morphology of ulcers.

Figure 22b shows that VAE achieves a higher reconstruction error (MSE = 0.011) and SSIM = 0.667. The heatmap of the residual values serves well to differentiate between the ulcer center and the healthy skin, proving the discriminatory capability of the model. Latent-space interpolation shows that structural changes in lesion intensity levels are consistent across a value, and supports the claim that the VAE learns a semantically meaningful latent space that is beneficial to further CL phase and classification. The reconstruction and residual analyses presented in Figs. 22 demonstrate that the VAE is able to capture global geometry of wound and point to local variations. The mean-squared error of reconstruction (MSE [? ] 0.012 +- 0.001) and structural similarity index (SSIM [? ] 0.54 +- 0.12) in diverse samples indicates a mediocre but a reliable quality of reconstruction that is typically portrayed by medical VAEs trained on limited datasets. The residual heatmaps highlight the regions where significant reconstruction error is spatially congruent with ulcer boundaries, confirming that the VAE serves as an implicit anomaly detector. In addition, the interpolations in latent space suggest a manifold that allows smooth morphing and, therefore, represents powerful continuous embeddings that enhance the discriminative capability of the subsequent DenseVAE-CL classifier.

### Visualization of classification results

**Fig. 23:**
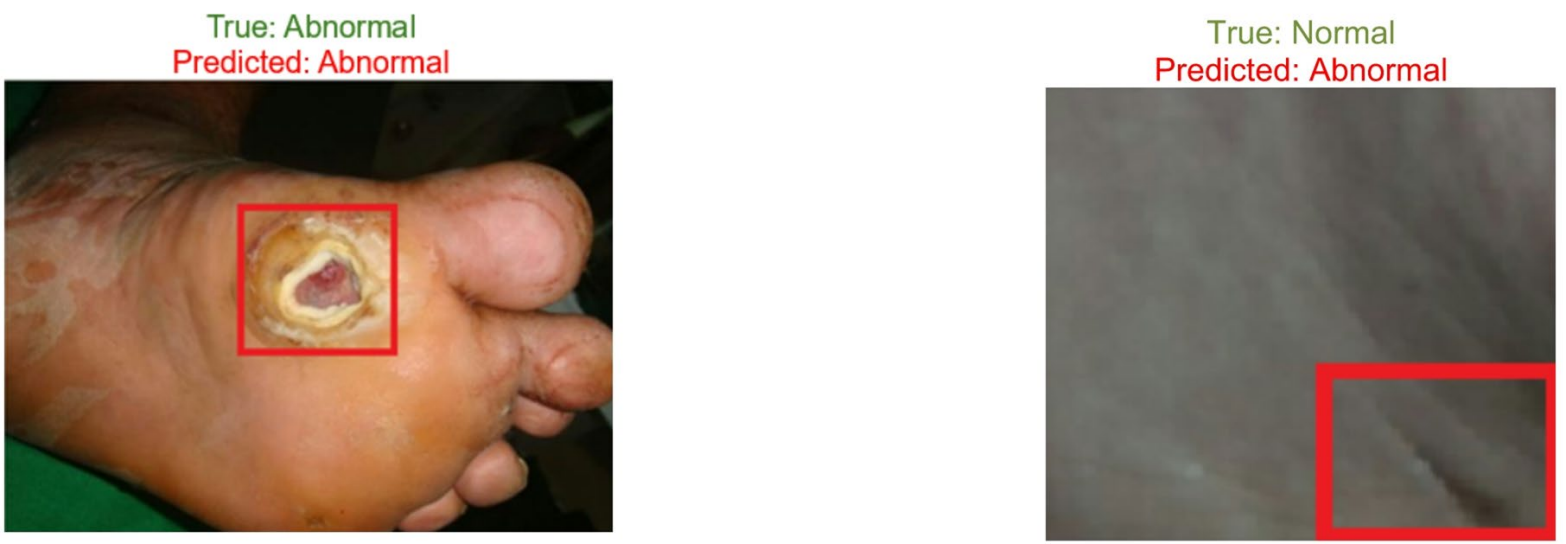
Visualization of image classification results.

The results of classification were presented in a systematic manner through visualization tools, which helped to understand the performance of the model. Figure 23 shows the result of the classification of the suggested model, which was received when testing the model against the previously unknown data. The case of a correctly detected ulcer is presented in Fig. 23a, and the case of a false diagnosis when the model predicted a case as normal rather than as an abnormal case is presented in Fig. 23b. Such mistakes mainly occur in cases where there is uneven lighting, partial obstruction due to ulcers, or in cases where skin patterns cover up the areas of the lesions.

### Grad cam analysis

**Fig. 24:**
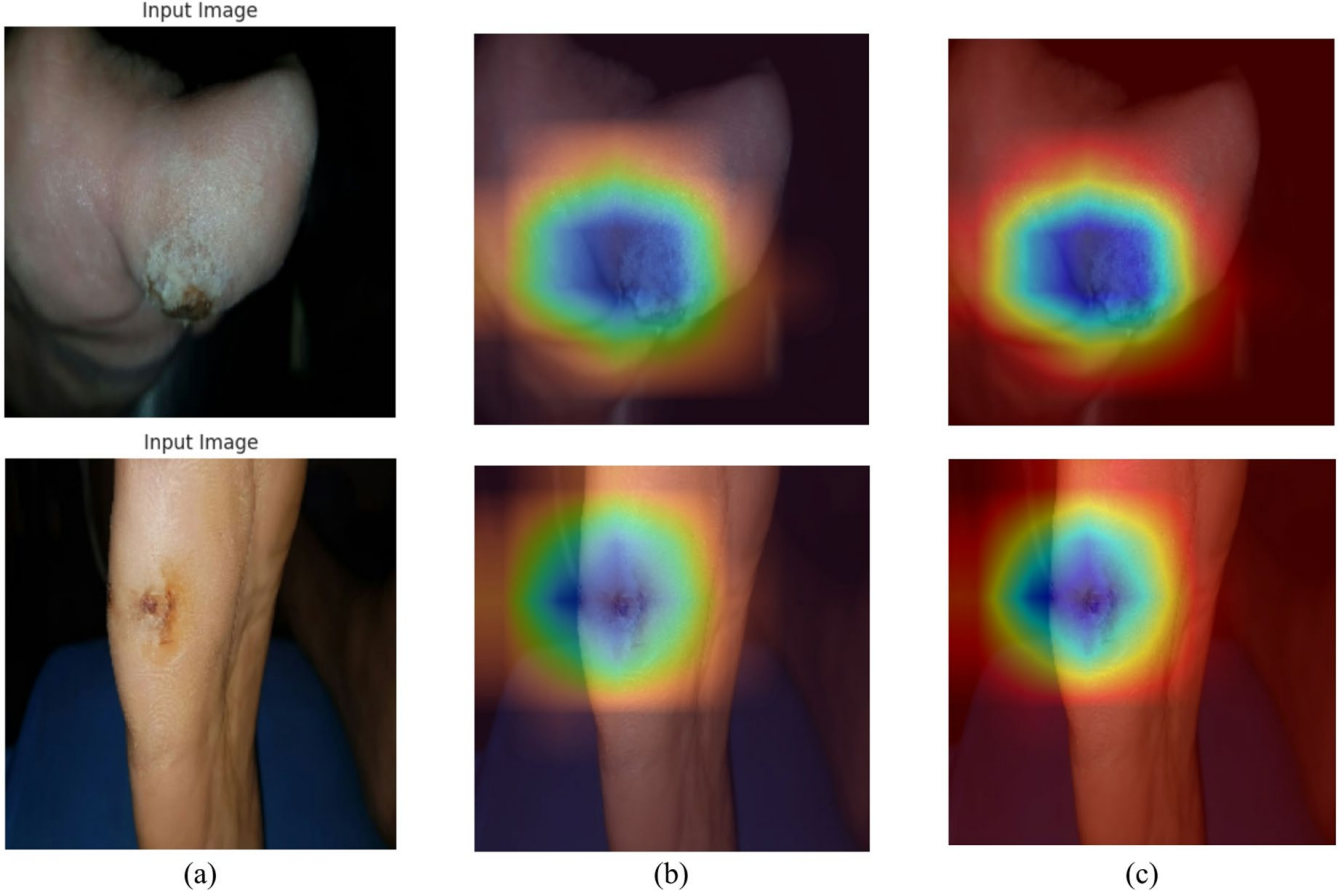
Grad-cam visualization of results (**a**) original image (**b**) CL grad-cam analysis.

Figure 24 showcases the Grad-CAM visualizations that contrast the CL-only model with the suggested DenseVAE-CL model. The input images shown in Fig. 24a displays typical DFU cases featuring different ulcer shapes and textures. The CL-only heatmaps shown in Fig. 24b displays wider and more dispersed activation, occasionally reaching nearby skin areas. In comparison, the DenseVAE-CL model generates clearer and more targeted attention aimed specifically at ulcer edges and central lesion areas which can be seen from Fig. 24c. The enhanced and more intense red–yellow activation in the VAE-CL maps signifies better interpretability and a more significant emphasis on key features. These comparisons show that including synthetic data generated by VAE assists the model in enhancing its focus and depending on clinically significant ulcer areas instead of background details.

(c) VAE-CL Grad-Cam Analysis.

## State-of-the-Art analysis

In order to assess the performance of the proposed DenseVAE-CL model, a comparative analysis was done with several state-of-the-art methods currently used to classify diabetic foot ulcer (DFU) using a publicly available dataset DFU Kaggle (2,673 images). Table 6 represents the key findings. The previous CTREE decision-tree algorithm^16^ was an interpretable but shallow learning approach with 0.795 accuracy, 0.806 sensitivity, 0.783 specificity and 0.88 AUC, but was weak at detecting complex visual patterns. The use of the DL models significantly increased performance. DFootNet^28^ used a dense neural network that included attention and achieved an accuracy of 0.988 and AUC of 0.981, and DFUXAI^29^ enhanced interpretability by adding SHAP, LIME, and Grad-Cam that had a ResNet50 backbone and achieved 0.9875 accuracy and 0.985 AUC. DFUXAI 2.0^30^ was the first model which used CNNs together with a Siamese Neural Network (SNN) to learn similarity-based features, with an accuracy of 0.9876 and an AUC of 0.986. Comparatively, the proposed DenseVAE-CL achieved the best overall performance with accuracy, precision, recall and F1-score, specificity, sensitivity and AUC being 0.996, 0.995, 0.994, 0.995, 1.000 and 0.990 respectively. These improvements can be credited to the additions of VAE-based synthetic data generation, CL to increase the inter-class difference and SE modules to recalibrate channels dynamically. The combination of these factors enhances the difference between features and model generalization among DFU appearances.

**Table 6:** State of the Art Analysis.

Ref/Year	Model/Technique	Dataset/ Images	Classes	Performance
^16^/2023	CTREE Decision Tree Algorithm for DFU Risk Prediction	DFU Kaggle/2673	2	Accuracy – 0.795 Sensitivity – 0.806 Specificity – 0.783 AUC – 0.88
^29^/2024	DFootNet	DFU Kaggle/2673	2	Accuracy-0.988 Precision-0.990, Recall-0.987 F1-score-0.988 AUC-ROC- 0.981
^30^/2024	DFU_XAI (Explainable Deep Learning Framework using SHAP, LIME, and Grad-CAM) — ResNet50 backbone	DFU Kaggle/2673	2	Accuracy – 0.9875 Precision – 0.992 Recall – 0.976 F1–0.984 AUC – 0.985
^31^/2025	DFU_XAI 2.0 (XAI integrating CNNs + Siamese Neural Network with SHAP, LIME, and Grad-CAM)	DFU Kaggle/2673	2	Accuracy – 0.9876 Precision – 0.993 Recall – 0.977 F1–0.985 AUC – 0.986
Proposed DenseVAE-CL Model		**DFU Kaggle/2673**	**2**	**Accuracy-99.60** **Precision-99.51** **Recall-99.41** **F-1–99.46** **AUC- 0.99** **Specificity-1.00** **Sensitivity- 0.989**

This rule to explainable and hybrid generative-contrastive deep network evolution evidently illustrates the advancement of DFU modeling and provides DenseVAE-CL with a new standard of accurate, robust, and interpretable DFU image classification.

## Conclusion

The study presents a powerful DL architecture of the automated Diabetic foot Ulcer (DFU) detection using a modified version of DenseNet121 with Variational Autoencoders (VAE) and Contrastive Learning. The DenseVAE-CL model used shows excellent performance, with accuracy of 99.6%, precision of 99.51% and recall of 99.41%, far exceeding existing methods. By relying on VAE to obtain latent features and CL to enhance the ability of class distinction, the model ensures proper classification of complex instances of DFU. Also the class-wise confidence calibration analysis shows that the mean confidence of the case of ulcers and the healthy skin are 0.9031 and 0.9692 respectively, and the Expected Calibration Error (ECE) of the two cases are 0.0457. This demonstrates the reliability of the model and its precisely predicted forecasts can support clinical decision-support systems. Overall, the framework can assist automated DFU image classification in research and screening settings, reducing diagnostic delays and complications. Although the proposed DenseVAE-CL framework has a high generalization, high interpretability and calibration does not fail in the 5-fold experiments, there are some limitations involved. The model requires a fairly large dataset, and even though synthesis based on VAE creates more diverse data, the generated samples are still not as realistic as GAN ones do. Further research will focus on the use of multi-institutional DFU data and integration of hybrid generative methods to enhance realism and clinical strength. In spite of these limitations, the framework demonstrates high diagnostic accuracy and interpretability, which proves that it can become a valuable and trusted AI-assisted diagnostic system.

## Data Availability

https://www.kaggle.com/datasets/laithjj/diabetic-foot-ulcer-dfu
